# Interplay of Obesity, Ethanol, and Contaminant Mixture on Clinical Profiles of Cardiovascular and Metabolic Diseases: Evidence from an Animal Study

**DOI:** 10.1007/s12012-022-09738-6

**Published:** 2022-04-16

**Authors:** Maria Florian, Bai Li, Dominique Patry, Jocelyn Truong, Don Caldwell, Melanie C. Coughlan, Robert Woodworth, Jin Yan, Qixuan Chen, Ivan Petrov, Laziyan Mahemuti, Michelle Lalande, Nanqin Li, Laurie H. M. Chan, William G. Willmore, Xiaolei Jin

**Affiliations:** 1 Regulatory Toxicology Research Division, Bureau of Chemical Safety, Food Directorate, HPFB, Health Canada, Ottawa, ON Canada; 2 Departments of Biology and Chemistry, Institute of Biochemistry, Carleton University, Ottawa, ON Canada; 3 Department of Biology, University of Ottawa, Ottawa, ON Canada; 4 Scientific Services Division, Bureau of Chemical Safety, Food Directorate, HPFB, Health Canada, Ottawa, ON Canada; 5 Hazard Identification Division, Environmental Health Science and Research Bureau, HECSB, Health Canada, Ottawa, ON Canada

**Keywords:** Obesity, Ethanol, Contaminants, Cardiovascular and metabolic diseases, Clinical markers

## Abstract

Obesity, ethanol, and contaminants are known risk factors of cardiovascular and metabolic diseases (CMD). However, their interplay on clinical profiles of these diseases remains unclear, and thus were investigated in this study. Male lean or obese JCR rats were given water or 10% ethanol and orally treated with or without a contaminant mixture (CM) dissolved in corn oil and loaded on two cookies at 0, 1.6, or 16 mg/kg BW/day dose levels for 4 weeks. The CM consisted 22 environmental contaminants found in human blood or serum of Northern populations. Over 60 parameters related to CMD were examined. The results revealed that obesity in JCR rats resembles the clinical profiles of non-alcoholic fatty liver disease in humans. Obesity was also associated with increased serum and organ retention of mercury, one of the chemical components of CM. Exposure to ethanol lightened hyperlipidemia, increased liver retention of mercury, and increased risk for hypertension in the obese rats. CM lessened hyperlipidemia and hyperenzymemia, worsened systemic inflammation and increased the risk for hypertension in the obese rats. CM markedly increased serum ethanol levels with or without ethanol exposure. Tissue total mercury contents significantly correlated with clinical parameters with altered profiles by both ethanol and obesity. These results suggest that obese individuals may be more prone to contaminant accumulation. Ethanol and CM exposure can alter clinical profiles associated with obesity, which may lead to misdiagnosis of CMD associated with obesity. CM can alter endogenous production and/or metabolism of ethanol, further complicating disease progression, diagnosis, and treatment.

**Supplementary Information:**

The online version contains supplementary material available at 10.1007/s12012-022-09738-6.

## Introduction

Cardiovascular diseases (CVDs) are the number one cause of death worldwide, with an estimate of 17.9 million loss of lives each year (WHO 2021, https://www.who.int/health-topics/cardiovascular-diseases). Atherosclerosis is the main underlying cause of CVDs that is characterized by hypercholesterolemia, hypertriglyceridemia, vascular inflammation, platelet activation, and thrombosis [1]. Dyslipidemia, immune cell activation, and endothelial dysfunction are the key processes involved in the initiation and progression of atherosclerosis [2–5]. Various biomarkers reflecting changes in these processes have been used in risk stratification, prevention, and treatment of CVDs [6–12].

Many modifiable and non-modifiable factors including diet, lifestyle, age, and genetic background can contribute to the pathogenesis of CVD. Obesity, either acquired or genetic, is a well-known risk factor for CVD [13, 14]. Non-alcoholic fatty liver disease (NAFLD), associated with obesity, is often linked to CVD outcome [15, 16]. Evidence also supports that heavy chronic alcohol consumption increases the risk of CVDs [17–20]. Systemic arterial hypertension has a strong, continuous positive association with CVDs [21].

Both experimental and epidemiological studies have demonstrated that environmental contaminants such as heavy metals, polychlorinated biphenyls (PCBs), and organochlorine pesticides are potential risk factors for obesity, diabetes, and CVDs [22–26], although the underlying mechanisms are not fully understood. Serum PCBs levels were found to be associated with elevated serum triglyceride [27] and cholesterol levels [28], increased blood pressure [29, 30], and higher rates of coronary heart disease and myocardial infarction in exposed populations [31]. Elevated methylmercury (MeHg) exposure was associated with increased diastolic and systolic blood pressure [32–35]. In experimental animals, MeHg decreased circulating levels of paraoxonase-1 (PON1), the major anti-atherosclerotic component of high-density lipoprotein (HDL) [36]. It also increased serum levels of oxidized low-density lipoprotein (Ox-LDL) that are known to contribute to inflammation and the formation of plaque. In addition, MeHg also increased serum monocyte chemotactic protein-1 (MCP-1), a cytokine known to be involved in monocyte infiltration and atherosclerosis. These effects of MeHg implies an increased risk of CVDs in exposed animals [36].

Some circumpolar populations have an increased prevalence of obesity, hypertension, diabetes, and/or CVD [37–39]. These chronic diseases have also been associated in the scientific literature with elevated levels of various environmental contaminants, alcohol use, and dietary shift from traditional to non-nutrient-dense foods [40–42]. However, there is a lack of understanding how multiple risk factors such as ethanol consumption, obesity, and contaminant exposure may interplay on the pathogenic processes and outcomes of these chronic diseases, especially CVDs. In a previous study, we investigated the effects and interactions of obesity, ethanol (EtOH), and a contaminant mixture (CM) on liver cholesterol homeostasis and energy metabolism in male obese and lean JCR rats. The rats were orally exposed to a CM containing 22 organic and inorganic environmental contaminants found in human blood or serum of highly exposed Northern populations at three dose levels, 0, 1.6, or 16 mg CM/kg BW/day, with or without co-exposure to 10% ethanol in drinking water [43]. Our results suggested that obesity in JCR rats was associated with hepatosteatosis, while EtOH exposure worsened the hepatosteatosis associated with obesity [43]. The CM exacerbated the existing hepatosteatosis in the obese rats, with or without co-exposure to EtOH [43].

In this study, we determined the effects of CM, EtOH, and obesity, alone and in combination, on over sixty clinical and physiological parameters related to risks and diagnosis of CMD in JCR rats.

## Materials and Methods

### Animal Model

All animal work was conducted according to the guideline of Canadian Council on Animal Care (CCAC). The protocol (HCO-ACC-2010-020) was approved by Health Canada Ottawa Animal Care Committee (HCO-ACC). All animals were housed, serologically tested, and euthanized as described previously [43, 44].

The lean (+/?) and obese (cp/cp) JCR:LA male rats at age of 8 weeks were obtained from the Metabolic and Cardiovascular Diseases Laboratory at the University of Alberta, Edmonton, Alberta, Canada. The cp/cp rats carry the autosomal recessive cp gene, resulting from the Tyr763Stop mutation for the leptin receptor (ObR). Without ObR, these rats are hyperleptinemic, and become obese, hyperlipidemic, and hyperinsulinemic [45]. The heterozygous+/cp or homozygous+/+rats (collectively known as+/? rats) are lean and metabolically normal, and thus serve as controls for the cp/cp rats.

### CM Composition and Concentration

The CM is a mixture of 22 inorganic and organic contaminants including heavy metals, organochlorines, polychlorinated biphenyls, perfluorinated compound, chlorophenols, and brominated flame retardant that were frequently detected at high concentrations in blood or serum samples from Inuit residing in Northern Canada (Table S1). Three contaminant dose levels were used in this study (Tables 1, S1), including (a) zero dose that was the vehicle control, (b) the low dose at 1.6 mg/kg BW that was the highest concentrations of contaminants detected in human blood or serum samples of Inuit populations in Northern Canada [46], and (c) the high dose at 16 mg/kg BW that was tenfold of the low dose. The values of the low and high doses of CM were obtained by summing up the concentrations of 22 chemical components included in the mixture (Table S1). These dose levels were chosen in hoping to achieve serum concentrations of contaminants in dosed JCR rats similar to those found in highly exposed Northern populations. CM stock solutions were prepared by dissolving organic chemicals in corn oil, inorganic chemicals in distilled and deionized water, and Perfluorooctane Sulfonate (PFOS) in acetone. Corn oil, water, and acetone, with or without the CM, were added on two Teddy Graham cookies produced by Christie (Toronto, Canada).

**Table 1:** Experimental groups used in the study

Rats	wk3	wk4	wk5	wk6	wk7	wk8	wk9	wk10	Group
18 lean rats	Basal diet + water + 2 blank cookies		6 rats: basal diet + water + 2 blank cookies		6 rats: diet + water + 2 cookies loaded with corn oil				LWV
			12 rats: basal diet + EtOH 1% to 10% + 2 blank cookies		6 rats: diet + EtOH 10% + 2 cookies with corn oil				LEV
					6 rats: diet + EtOH 10% + 2 cookies with 1.6 mg CM/kg BW				LEL
48 obese rats	Basal diet + water + 2 blank cookies		24 rats: basal diet + water + 2 blank cookies		8 rats: diet + water + 2 cookies with corn oil				OWV
					8 rats: diet + water + 2 cookies with 1.6 mg CM/kg BW				OWL
					8 rats: diet + water + 2 cookies with 16 mg CM/kg BW				OWH
			24 rats: basal diet + EtOH 1% to 10% + 2 blank cookies		8 rats: diet + EtOH 10% + 2 cookies with corn oil				OEV
					8 rats: diet + EtOH 10% + 2 cookies with 1.6 mg CM/kg BW				OEL
					8 rats: diet + EtOH 10% + 2 cookies with 16 mg CM/kg BW				OEH

JCR obese and lean rats were acclimatized on AIN93G diet for 2 weeks (wks) before going through various treatments as shown above

### Animal Treatment and Sample Collection

As described in our previous study [44] and illustrated in Table 1 and Fig. S1, 48 obese and 18 lean rats were fed an AIN93G diet (Harlan Laboratory, Madison, WI, USA) and acclimatized for 2 weeks, and then fed the same diet and provided additional two cookies with or without (vehicle control) the CM per rat per day for 8 weeks. From the third week of the study, the lean and obese rats were each randomly divided into two groups. One group was given water ad libitum for a total of 6 weeks. The other group was provided 1% EtOH in drinking water on the first day, followed by 1% increment every other day until 10% EtOH in drinking water in a period of two weeks. Then, the 10% EtOH was continued for another 4 weeks until the end of study. From the fifth week of the study, the lean rats given EtOH and obese rats given either water or EtOH were randomly divided again into three groups (6 or 8 rats per group) that were dosed daily by giving corn oil (vehicle control), 1.6 mg CM/kg BW/day, or 16 mg CM/kg BW/day, respectively, on two adulterated cookies for a total of 4 weeks until the end of the study. Since the lean rats avoided eating cookies containing high-dose CM, only the vehicle and low-dose CM groups were used for lean rats. The amount of vehicle or CM loaded on cookies was calculated based on rat body weight measured the day before dosing. Dosing cookies were prepared on the night before doing and air dried to evaporate acetone and water. Consumption of cookies were monitored to ensure intended doses being achieved. At the end of the study, animals were exsanguinated via the abdominal aorta under isoflurane anesthesia. Isoflurane (AErrane, USP, Baxter Corporation Mississauga, Ontario, Canada) was used at a concentration of 5% with 1.5 L oxygen/min by inhalation for about 3–5 min. Organs were collected and kept frozen in liquid nitrogen. Blood samples were collected for hematology, clinical biochemistry and biomarker analysis. Food consumption (F-C) and water or EtOH consumption (W/E-C) were measured weekly.

Conditions of all 9 treatment groups are summarized in Table 1. The groups are designated using combination of three letters, with the first as L or O for lean or obese, respectively, the second as W or E for water or EtOH, respectively, and the third as V, L, or H for vehicle, low-dose CM, or high-dose CM, respectively, as shown in Table 1, with LWV for lean rats given water and treated with vehicle; LEV and LEL for lean rats given EtOH and treated with vehicle and low-dose CM, respectively; OWV, OWL, and OWH for obese rats given water and treated with vehicle, low-dose CM, and high-dose CM, respectively; and OEV, OEL, and OEH for obese rats given EtOH and treated with vehicle, low-dose CM, and high-dose CM, respectively. In correlation analysis, LEVL is for combined group of LEV and LEL, OWVL for combined group of OWV and OWL, OEVL for combined group of OEV and OEL.

### Heart Weight and Histopathology

Hearts were dissected, drained, and weighed. Hearts were then fixed in 10% buffered formalin for histology. Fixed hearts were embedded in paraffin wax. Sections of 5 µm thickness were cut on microtome, deparaffinized, and stained with hematoxylin and eosin. Pathological changes were examined under light microscope with a ×10 objective. Heart lesions were recorded following a developed protocol for the JCR:LA-cp rat [45]. Degree of lesions were graded from 1 to 5 with the lowest at 1 and the highest at 5. Certain types of lesions including fat infiltration, myocyte degeneration, hemorrhage, fibrosis, granulation, and plague formation were also recorded. However, due to limited number of incidences found in heart sections, statistical analysis could not be made, and thus treatment effects could not be quantitatively compared.

### Serum Lipids, Lipoproteins, and Related Biomarkers

A panel of serum lipid and lipoprotein markers including TC, LDL-C, HDL-C, TG, and lipase were analyzed using a Pentra 400 clinical chemistry analyser (Horiba Medical, Irvine, CA, USA) with corresponding reagents according to manufacturer’s instructions. Ox-LDL was measured using Mercodia Oxidized LDL competitive ELISA kits from ALPCO Diagnostic (NH, USA) according to manufacturer’s instructions. PON1 was measured using an EnzChek Paraoxonase Assay Kit from Invitrogen (Molecular Probes Inc., OR, USA) as described in the kit insert. ApoA1 was measured using an ELISA kit from Cloud-Clone Corp (Katy, TX, USA) according to manufacturer’s instructions. PON1 and ApoA1 values were also normalized against HDL-C and expressed as ratio of PON1/HDL-C and ApoA1/HDL-C.

### Inflammatory and Hematological Parameters

Whole blood was collected into EDTA tube and analyzed for hematology using Beckman Coulter AcT 5 Diff Hematology Analyzer. The Act 5diff CP system employs absorbance cytochemistry and volume (AcV) technology. Monocyte, neutrophil, and eosinophil populations were identified, using the absorbance patterns produced by differential cytochemical staining of their granules versus volume. Lymphocytes remained unstained, and the basophil population was analyzed on a separate channel using volume gating and selective lysis. Platelet counts (PLT) and mean platelet volume (MPV) were also measured. Red blood cell parameters included red blood cell count (RBC), hematocrit (HCT), hemoglobin (HGB), mean corpuscular volume (MCV), mean corpuscular hemoglobin concentration (MCHC), and red blood cell distribution width (RDW). Only data showing significant differences or correlations with CM or total mercury content among treatment groups are presented in Results section. Ratios of neutrophil counts (NC) to monocyte counts (MC), lymphocyte counts (LC), eosinophil counts (EC), and basophil counts (BC) were calculated and designated as N/M-C, N/L-C, L/E-C, and L/B-C, respectively.

Serum CRP was measured using an ELISA kit from Alpha Diagnostic International (Cat. #1010) (TX, USA) as described previously [36]. Samples were diluted 7800 times in sample diluent prior to analysis. Serum MCP-1 was determined using a rat MCP-1 EIA kit from ALPCO Diagnostics (Cat# 45-MCPR-1011) (NH, USA) according to manufacturer’s protocol.

### Kidney Function Markers and Vascular Endpoints

Serum levels of creatinine, sodium (Na), potassium (K), magnesium (Mg), chloride (Cl), phosphorus (P), urea nitrogen (BUN), and uric acid (UA) were measured using a Pentra 400 clinical chemistry analyser (Horiba Medical, Irvine, CA, USA) with corresponding reagents according to instructions. Only the parameters that showed significant differences between groups are included in the Results.

Serum nitric oxide (NO) was measured using a colorimetric non-enzymatic assay kit from Oxford Biomedical Research (Cat# NB88) according to manufacturer’s instruction. Serum 6-keto prostaglandin F1α (6-keto-PGF1α) was measured using an EIA kit from Cayman Chemical Company (Cat# 515211) according to the kit insert.

### Liver Function Markers

Serum total protein (TP), albumin, alanine aminotransferase (ALT), aspartate aminotransferase (AST), alkaline phosphatase (ALP), deconjugated bilirubin (Bil-D), total bilirubin (Bil-T), and iron levels were measured using a Pentra 400 clinical chemistry analyser (Horiba Medical, Irvine, CA, USA) with corresponding reagents according to manufacturer’s instruction. Serum EtOH levels were analyzed using Ethanol Assay Kit from Abcam (Toronto, ON, Canada) according to kit insert.

### Other Metabolic and Tissue Injury Markers

Serum total creatine kinase (CK), amylase (Amy), glucokinase (GK), and lactate dehydrogenase (LDH), were measured using a Pentra 400 clinical chemistry analyser (Horiba Medical, Irvine, CA, USA) with corresponding reagents according to manufacturer’s instructions.

### Total Mercury Contents

Serum, liver, muscle, kidney, pancreas, hypothalamus/thalamus, cerebellum, cortex, and corpus callosum samples were analyzed for total mercury (tHg) using the Nippon MA3000 direct combustion mercury analyzer (Nippon North America, College Station, TX). Analytical accuracy and precision were monitored through the use of Standard Reference Materials (SRMs), and intermittent analysis of duplicate samples. SRMs included National Research Council of Canada (NRCC) DOLT-4 (dogfish liver) and DORM-3 (dogfish muscle). The mercury working standard was made by successive dilution of stock mercury solution of 1000 ppm (Wako Pure Chemical Industries, Ltd. Japan) in 0.001% l-cysteine solution to make 1 ppm mercury solution.

### Statistical Analysis

All statistical analyses were carried out using SigmaPlot 12.0 (Systat Software, Inc. San Jose, CA, USA). Statistical comparisons for parametric data were performed using one way ANOVA with Tukey’s post-hoc test or Dunn’s test for unequal group size. Confidence values were set to 95%. SigmaPlot automatically performs a normality test when running a statistical procedure that makes assumptions about the population parameters. If the data fails the assumptions required for a particular test, SigmaPlot will suggest the appropriate test that can be used instead. Shapiro–Wilk is the default test for normality. For parametric data, Pearson Product Moment correlation analysis (for parametric data) was performed for clinical parameters, CM dose, and tissue total Hg levels. Significant correlations were identified with *p* < 0.05. Non-parametric data were transformed before correlation analysis. For data failed normality test after transformation, Spearman Rank Order Correlation analysis was applied.

## Results

### Heart Weight and Histopathology

**Fig. 1:**
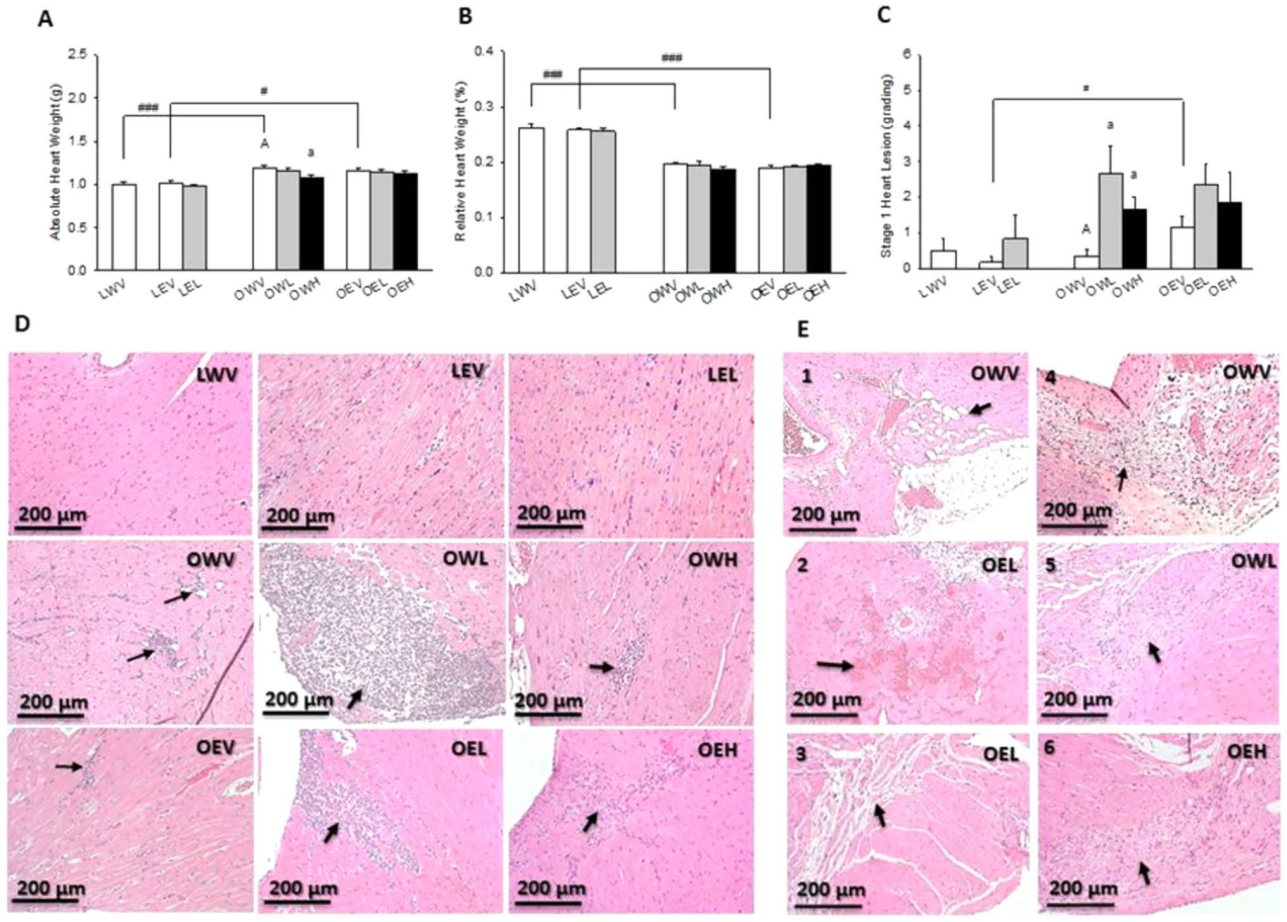
Effects of obesity, EtOH and CM on physiology and pathological parameters of heart, including absolute (**A**) and relative (**B**) heart weight, stage 1 heart lesion (**C**) and (**D**), and other histological lesions (**E**) observed in H&E staining of paraffin sections of heart organ under ×20 objective of a microscope (D-I). In **D**, black arrows indicate stage 1 lesion. In **E**, black arrow shows fat infiltration (E1), hemorrhage (E2), fibrosis (E3), granulation (E4, and E5) and plague formation (E6). Vertical bars represent the mean values from 4–8 rats. The error bars are the standard error of the means. “A” is significantly different from “a” at *p* < 0.05. “^#^” and “^###^” indicate significant differences between the two treatment groups located under the vertical lines at *p* < 0.05 and 0.001, respectively. *p* values were obtained from One Way ANOVA

As shown is Fig. 1, the absolute heart weights of rats in LWV and LEV groups were significantly lower (83.7% and 87.7%) than those in the OWV and OEV groups, with *p* = 0.013 and *p* < 0.001, respectively (Fig. 1A). However, the percentage relative heart weights of the rats in the LWV and LEV groups (or ratio of heart weight to body weight) were significantly higher (132.9% and 135.8%) than those in the OWV and OEV groups both with *p* < 0.001 (Fig. 1B). Absolute heart weights of the rats were significantly lower (90.4%) in the OWH than OWV group with *p* = 0.01 (Fig. 1A).

Histopathological examination revealed significantly higher degree of stage 1 heart lesion, defined as focal necrosis with chronic inflammatory cell infiltration, in the OEV (699.9%) than LEV group with *p* = 0.041 (Fig. 1C). The degree of the stage 1 heart lesion was significantly higher in the OWL (800.1%) and OWH (500.1%) groups than the OWV group with *p* = 0.014 and *p* = 0.007, respectively. An increased trend of the stage 1 heart lesion was also observed in the OEL and OEH groups as compared to OEV group, although it did not reach statistical significance. Typical microscopic images of the stage 1 lesion are shown in Fig. 1D. Other types of lesions including fat infiltration (Fig. 1E1), hemorrhage (Fig. 1E2), fibrosis (Fig. 1E3), granulation (Fig. 1E4 and E5), and formation of plaque like structure (Fig. 1E6) were also found in the obese rats, regardless of EtOH and CM. These lesions are known to be associated with myocardial infarction.

### Serum Lipids and Lipoproteins

**Fig. 2:**
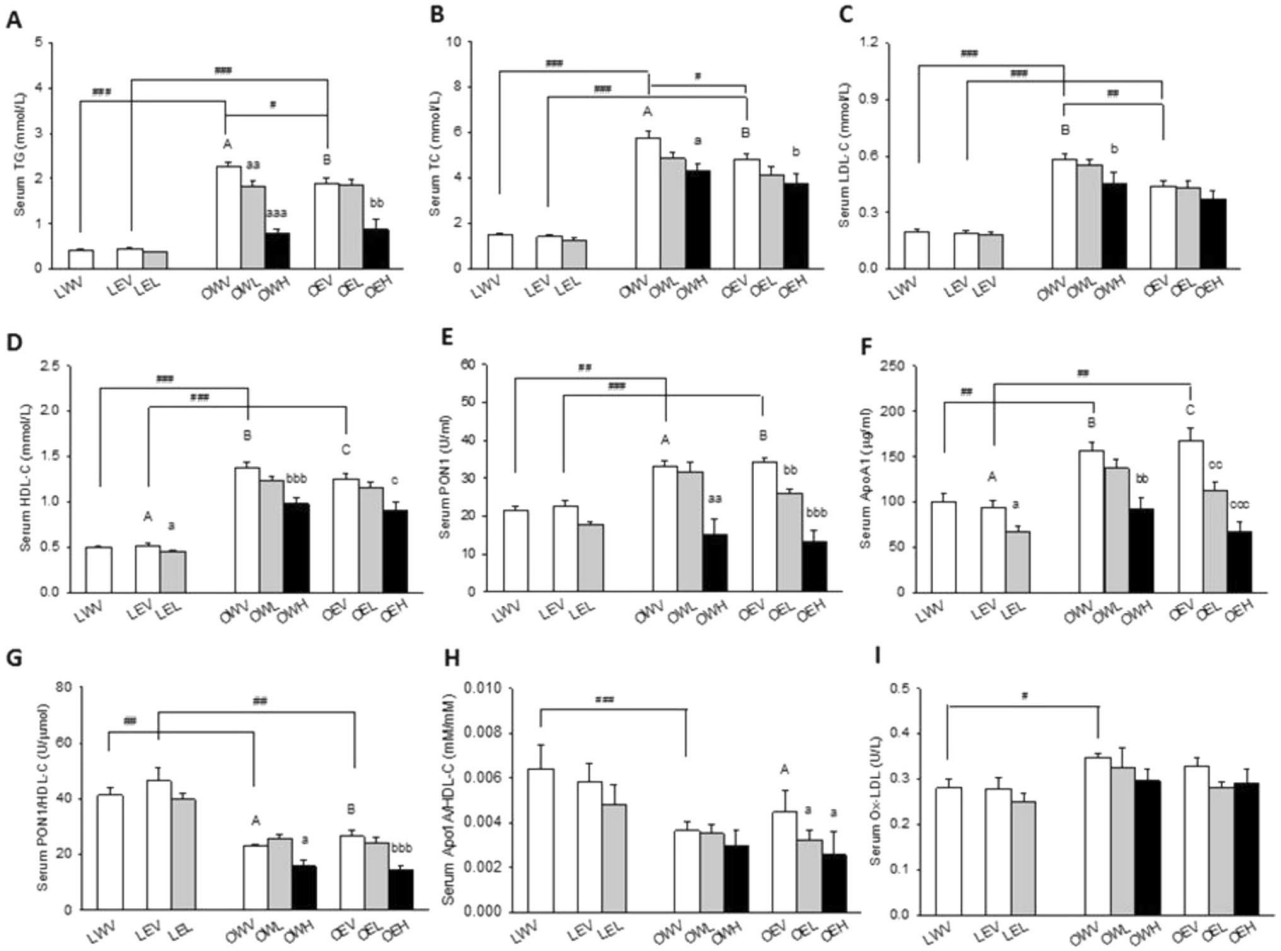
Effects of obesity, EtOH, and CM on circulating lipid and lipoprotein related markers. including serum triglycerides (TG) (**A**), total cholesterol (TC) (**B**), low-density lipoprotein cholesterol (LDL-C) (**C**), high-density lipoprotein cholesterol (HDL-C) (**D**), paraoxonase-1 (PON1) (**E**), apolipoprotein A1 (ApoA1) (**F**), ratio of PON1 to HDL cholesterol (**G**), ratio of ApoA1/HDL-C (**H**), and Ox-LDL (**I**). Vertical bars represent the mean values from 4–8 rats. The error bars are the standard error of the means. “A” is significantly different from “a”, “aa”, and “aaa” at *p* < 0.05, 0.01, and 0.001, respectively. “B” is significantly different from “b”, “bb”, and “bbb” at *p* < 0.05, 0.01, and 0.001, respectively. “C” is significantly different from “c”, “cc”, and “ccc” at *p* < 0.05, 0.01, and 0.001, respectively. “^#^”, “^##^”, and “^###^” indicate significant differences between the two treatment groups located under the vertical lines at *p* < 0.05, 0.01, and 0.001, respectively. *p* values were obtained from One Way ANOVA

The rats in the OWV (553.2%) and OEV (425.6%) groups had significantly higher levels of serum TG than those in the LWV and LEV groups, respectively, both with *p* < 0.001 (Fig. 2A), although the serum TG levels for the OEV group were significantly lower (82.2%) than those for the OWV group with *p* = 0.027. The rats in the OWL (74.8%) and OWH (31.5%) groups had significantly lower levels of TG than those in the OWV group with *p* = 0.007 and *p* < 0.001, respectively. The rats in the OEH group (46.9%) had significantly lower serum TG levels than those in the OEV group with *p* = 0.001. Similar trends were also observed for serum TC (Fig. 2B), LDL-C (Fig. 2C) and HDL-C (Fig. 2D) levels. More specifically, rats in the OWV (381.7%) and OEV (339.7%) groups had significantly higher serum TC levels than those in the LWV and LEV groups, respectively, with *p* < 0.001 for both comparisons (Fig. 2B). Rats in the OWH (74.9%) and OEH (78.4%) groups had significantly lower serum TC levels than those in the OWV and OEV groups with *p* = 0.01 and *p* = 0.027, respectively. Rats in the OWV (295.2%) and OEV (229.5%) groups had significantly higher LDL-C than those in LWV and LEV groups, respective, both with *p* < 0.001 (Fig. 2C). Rats in the OEV (75.2%) group had significantly lower LDL-C than those in the OWV group with *p* = 0.003. The rats in the OWV (274.7%) and OEV (240.9%) groups had significantly higher serum HDL-C levels than those in the LWV and LEV groups, respectively, both with *p* < 0.001. The rats in the OWH (70.9%), OEH (71.8%), and LEL (87.5%) groups had significantly lower serum HDL-C than those in the OWV, OEV, and LEV groups with *p* < 0.001, *p* = 0.039, and *p* = 0.015, respectively (Fig. 2D).

**Fig. 3:**
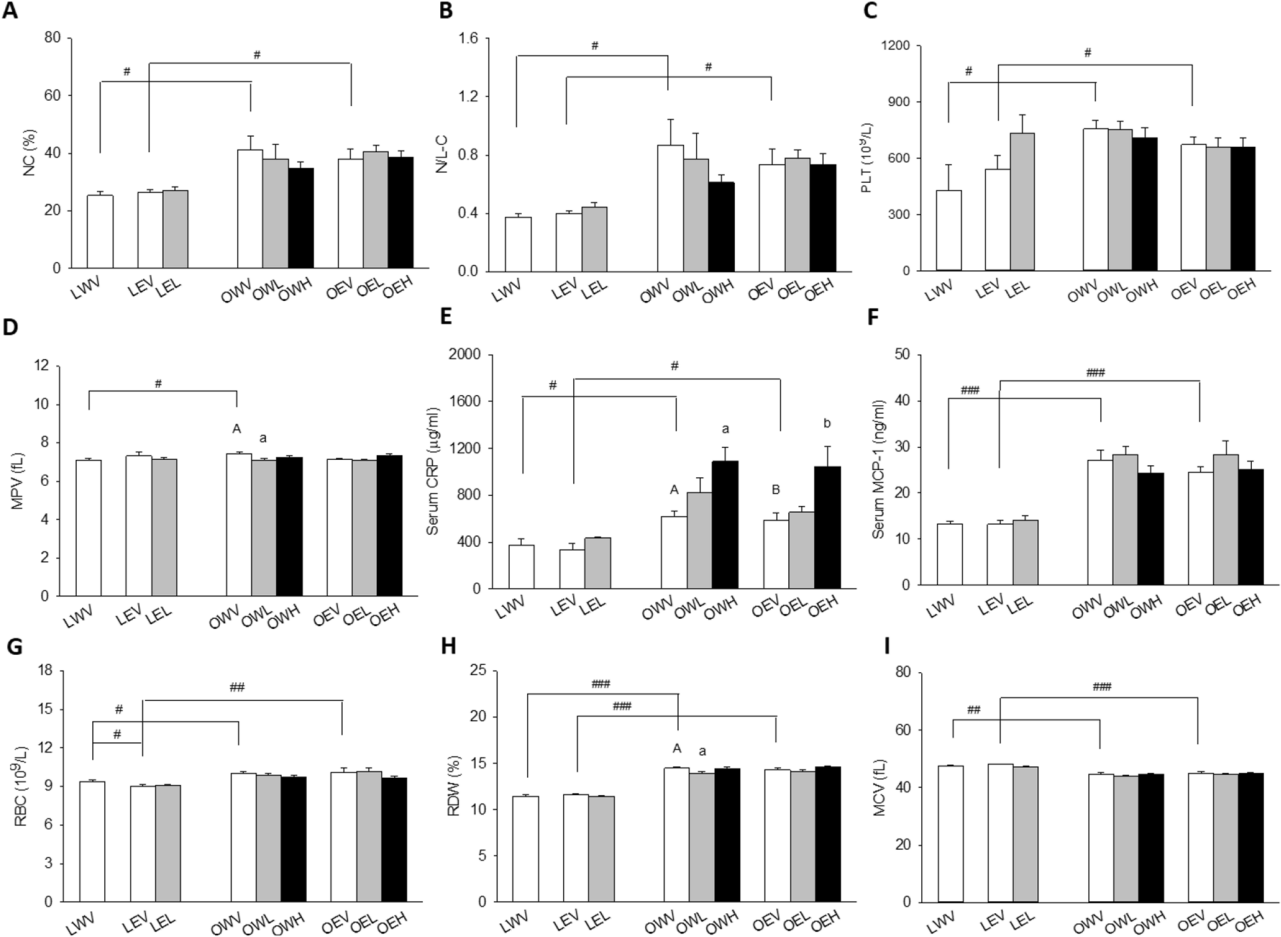
Effects of obesity, EtOH, and CM on inflammatory and hematological markers including neutrophil counts (NC) (**A**), neutrophil to lymphocytes ratio (N/L-C) (**B**), platelet counts (PLT) (**C**), mean platelet volume (MPV) (**D**), C-reactive protein (CRP) (**E**), monocyte chemoattractant protein-1 (MCP-1) (**F**), red blood cell counts (RBC) (**G**), red cell distribution width (RDW) (**H**), and mean corpuscular volume (MCV) (**I**). Vertical bars represent the mean values from 4–8 rats. The error bars are the standard error of the means. “A” is significantly different from “a” at *p* < 0.05. “B” is significantly different from “b” at *p* < 0.05. “^#^”, “^##^”, and “^###^” indicate significant differences between the two treatment groups located under the vertical lines at *p* < 0.05, 0.01, and 0.001, respectively. *p* values were obtained from One Way ANOVA

The rat serum PON1 levels were significantly higher in the OWV (154.5%) and OEV (151.9%) groups than LWV and LEV groups with *p* < 0.001 and *p* = 0.002, respectively (Fig. 2E). They were also significantly lower in the OEL (76.8%) and OEH (39.3%) than OEV groups with *p* = 0.004 and *p* < 0.001, respectively, and in the OWH (46.0%) than OWV group with *p* = 0.006. The serum PON1 levels were also lower in the LEL than LEV group, although not to statistically significant degrees. Similar to serum PON1, the levels of serum ApoA1 were significantly higher in the OWV (157.2%) and OEV (179.1%) than the LWV and LEV groups, respectively, both with *p* = 0.002. (Fig. 2F). Their levels were significantly lower in the LEL (81.8%) and OEL (64.2%) than LEV and OEV groups with *p* = 0.028 and *p* = 0.005, respectively, and further lower in the OWH (58.6%) and OEH (40.2%) groups than OWV and OEV groups with *p* = 0.001 and *p* < 0.001, respectively. Serum PON1 activity is known to be positively associated with serum HDL-C. We also calculated the ratios of PON1 to HDL-C and found them to be significantly lower in the OWV (49.7%) and OEV (57.1%) than the LWV and LEV groups with *p* = 0.001 and *p* = 0.008, respectively. They were further decreased in the OWH (68.2%) and OEH (54.8%) groups versus OWV and OEV groups with *p* = 0.027 and *p* = 0.003, respectively (Fig. 2G). Similarly, the ratios of ApoA1 to HDL-C were lower in the OWV (56.7%) than the LWV group with *p* < 0.001 and in the OEL (72.0%) and OEH (57.2%) groups than the OEV group with *p* = 0.041 and *p* = 0.021, respectively (Fig. 3H). The serum levels of Ox-LDL was significantly higher in the OWV (123.8%) than LWV group with *p* = 0.016 (Fig. 2I), while there was no significant differences among other groups.

### Inflammatory and Hematological Parameters

**Fig. 4:**
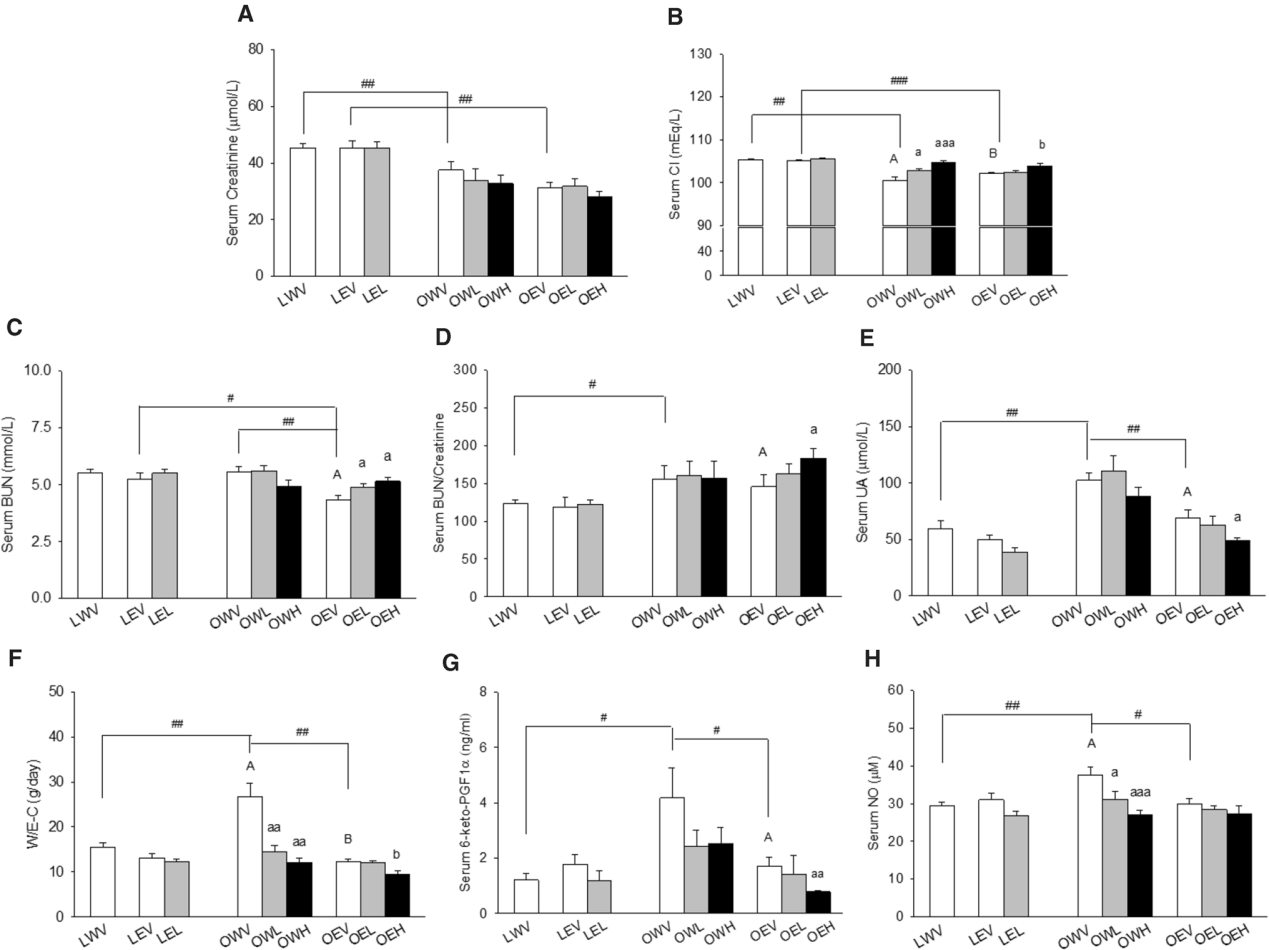
Effects of obesity, EtOH, and CM on clinical markers related to kidney function and blood pressure regulation including serum creatinine (**A**), chloride (Cl) (**B**), blood urea nitrogen (BUN) (**C**), BUN/creatinine ratio (**D**), uric acid (UA) (**E**), water or EtOH consumption (W/E-C) (**F**), 6-keto-prostaglandian F1α (6-keto-PGF1α) (**G**), and nitric oxide (NO) (**H**). Vertical bars represent the mean values from 4–8 rats. The error bars are the standard error of the means. “A” is significantly different from “a”, “aa”, and “aaa” at *p* < 0.05, 0.01, and 0.001, respectively. “B” is significantly different from “b” at *p* < 0.05. “^#^”, “^##^”, and “^###^” indicate significant differences between the two treatment groups located under the vertical lines at *p* < 0.05, 0.01, and 0.001, respectively. *p* values were obtained from One Way ANOVA

The obese rats had elevated levels of a number of inflammatory, thrombotic, and anisocytosis markers in the circulation as compared to lean rats (Fig. 3). The rats in the OWV (162.1% for NC, 231.5% for N/L-C, and 176.4% for PLT) and OEV (143.4% for NC, 183.9% for N/L-C, 123.8% for PLT) groups had significantly higher NC (Fig. 3A), N/L-C (Fig. 3B), and PLT (Fig. 3C) than those in the LWV and LEV groups with *p* = 0.035 and *p* = 0.026 for NC, *p* = 0.035 and *p* = 0.028 for N/L-C, and *p* = 0.016 and *p* = 0.013 for PLT, respectively. The rats in the OWV (104.9%) group had significantly higher levels of MPV than those in the LWV group with *p* = 0.035 (Fig. 3D). The serum CRP levels were significantly higher in the OWV (163.9%) and OEV (175.4%) groups than LWV and LEV groups with *p* = 0.013 and *p* = 0.021, respectively, and doubled in the OWH (176.4%) and OEH (176.8%) groups versus OWV and OEV groups, with *p* = 0.01 and *p* = 0.05, respectively (Fig. 3E). The rats in the OWV (203.8%) and OEV (185.6%) groups had significantly higher serum levels of MCP-1 than those in the LWV and LEV groups, respectively, both with *p* < 0.001, while CM treatment had no influence on this parameter (Fig. 3F). The rats in the OWV (105.3% for RBC, 126.3% for RDW, 93.6% for MCV) and OEV (112.1% for RBC, 123.6% for RDW, 89.8% for MCV) groups also had higher RBC (Fig. 3G) and RDW (Fig. 4H) and lower MCV (Fig. 3I) than those in the LWV and LEV groups, with *p* = 0.01 and *p* = 0.005 for RBC, *p* < 0.001 and *p* < 0.001 for RDW, *p* = 0.002 and *p* < 0.001 for MCV, respectively. The rats in the LEV group had significantly lower RBC (96.0%) than those in the LWV group with *p* = 0.029 (Fig. 3G). The rats in the OWL (96.4%) group had significantly lower RDW than those in the OWV group with *p* = 0.023 (Fig. 3I).

### Kidney Function Markers and Blood Pressure Regulators

The rats in the LWV (120.7%) and LEV (145.7%) groups had significantly higher serum creatinine levels than those in the OWV and OEV groups with *p* = 0.038 and *p* < 0.001, respectively (Fig. 4A). Neither EtOH nor CM, had significant influence on this parameter. The rats in the LWV (104.7%) and LEV (102.9%) groups also had significantly higher serum Cl levels than those in the OWV and OEV groups, with *p* = 0.001 and *p* < 0.001, respectively (Fig. 4B). The serum Cl levels were elevated in the OWL (102.2%) group versus OWV group with *p* = 0.032, and further elevated in the OWH (104.1%) and OEH (101.7%) groups versus the OWV and OEV groups, with *p* < 0.001 and *p* = 0.042, respectively. Serum BUN levels were significantly higher in the LEV (120.3%) and OWV (128.0%) groups than the OEV group with *p* = 0.026 and *p* = 0.002, respectively (Fig. 4C). The rats in the OEL (112.6%) and OEH (118.4%) groups had significantly higher levels of serum BUN than those in the OEV group with *p* = 0.041 and *p* = 0.011, respectively (Fig. 4C). The ratio of BUN/creatinine, often used to evaluate kidney injury, was significantly higher in the OWV (134.4%) than the LWV group with *p* = 0.049, and OEH (125.4%) than the OEV group, with *p* = 0.028 (Fig. 4D). The serum UA levels were significantly higher in the OWV (171.4% and 148.1%) than the LWV and OEV groups with *p* = 0.002 and *p* = 0.004, respectively, and also higher in the OEV (140.3%) than OEH group with *p* = 0.026 (Fig. 4E).

Water consumption was significantly higher in the OWV (202.9% and 215.8%) than LWV and OEV groups with *p* = 0.05 and *p* = 0.008, respectively (Fig. 4F). The water consumption dropped to 54.4% in the OWL group versus the OWV group with *p* = 0.008, and dropped even further in the OWH (50.3%) group with *p* = 0.002. The EtOH consumption was significantly lower in the OEH (76.1%) than OEV group with *p* = 0.015. The serum 6-keto-PGF1α level was significantly higher in the OWV (344.5%) than the LWV group with *p* = 0.032. This parameter dropped significantly in the OEH (45.6%) versus OEV group with *p* = 0.008 (Fig. 4G). A trend of decrease was also observed in the OWL (58.2%) and OWH (60.8%) versus OWV groups, although the differences did not reach statistical significance. Similarly, serum NO levels were significantly higher in the OWV group than the LWV and OEV groups with *p* = 0.009 and *p* = 0.01, respectively (Fig. 4H). The serum NO levels were significantly lower in the OWL (82.8%) and OWH (72.2%) groups than the OWV group with *p* = 0.05 and *p* = 0.001, respectively.

### Liver Function Parameters

**Fig. 5:**
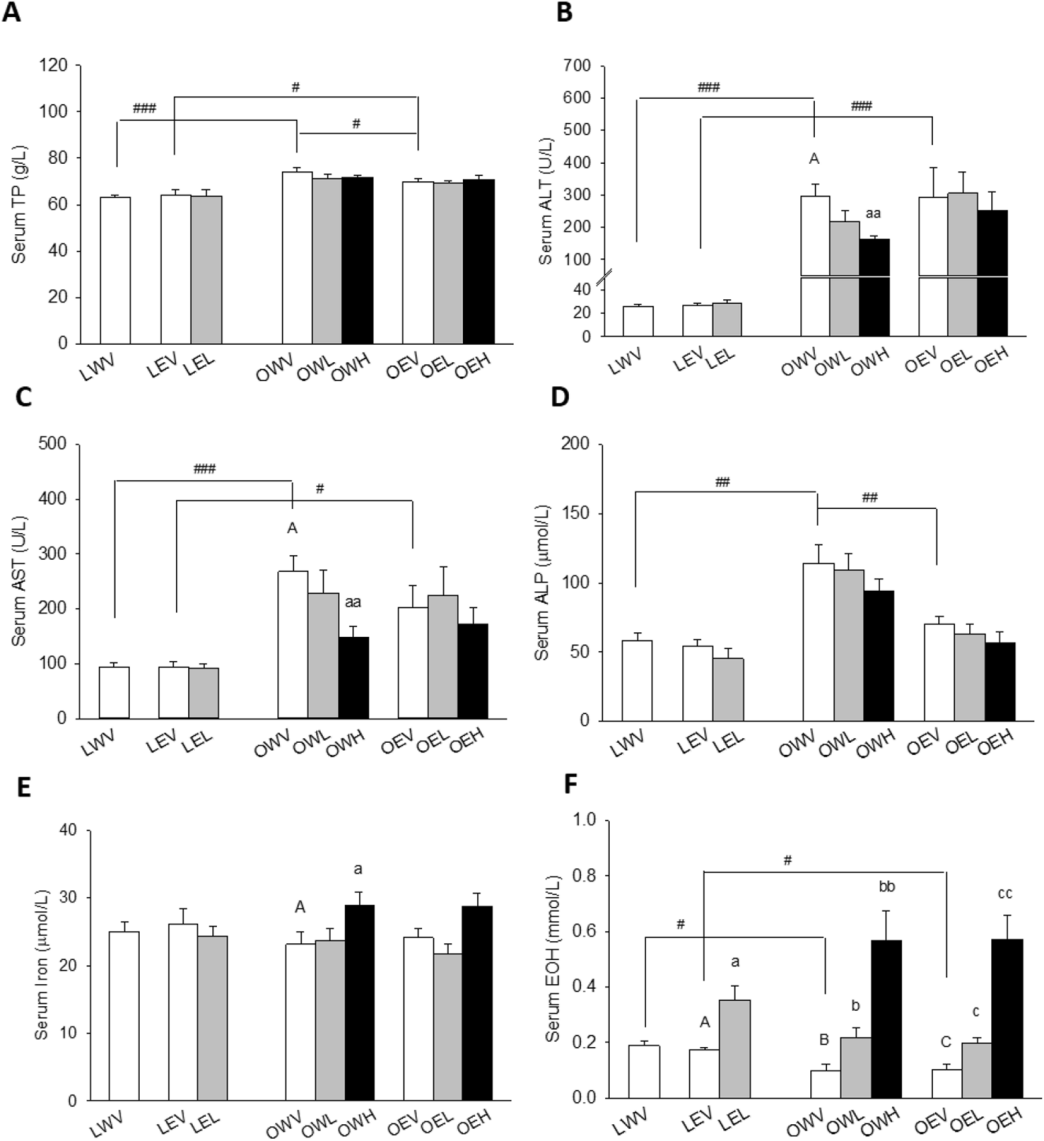
Effects of obesity, EtOH, and CM on liver function markers including serum protein (TP) (**A**), alanine aminotransferase (ALT) (**B**), aspartate aminotransferase (AST) (**C**), alkaline phosphatase (ALP) (**D**), iron (**E**), and EtOH (**F**). LW for lean rats given water. Vertical bars represent the mean values from 4 to 8 rats. The error bars are the standard error of the means. “A” is significantly different from “a” and “aa” at *p* < 0.05 and 0.01, respectively. “B” is significantly different from “b” and “bb” at *p* < 0.05 and 0.01, respectively. “C” is significantly different from “c” and “cc” at *p* < 0.05 and 0.01, respectively. “^#^”, “^##^”, and “^###^” indicate significant differences between the two treatment groups located under the vertical lines at *p* < 0.05, 0.01, and 0.001, respectively. *p* values were obtained from One Way ANOVA

Serum total protein levels were significantly higher in the OWV (117.9%) and OEV (108.8%) than the LWV and LEV groups with *p* < 0.001 and *p* = 0.025, respectively, and also higher in the OWV (106.2%) group than OEV group with *p* = 0.03 (Fig. 5A). Serum ALT levels were over tenfold higher in the OWV (1130.6%) and OEV (1096.3%) groups than LWV and LEV groups, respectively, both with *p* < 0.001, and dropped significantly in the OWH (54.9%) versus the OWV group with *p* = 0.007 (Fig. 5B). Similar to serum ALT, serum AST levels were significantly higher in the OWV (286.5%) and OEV (215.6%) groups than the LWV and LEV groups with *p* < 0.001 and *p* = 0.013, respectively, and significantly lower in the OWH (55.2%) than the OWV group with *p* = 0.005) (Fig. 5C). Serum ALP levels were significantly higher in the OWV than LWV and OEV groups with *p* = 0.001 and *p* = 0.002, respectively. Although a trend of decrease in serum ALP levels with CM dose was observed in obese rats given either water or EtOH, none of them reached statistical significance with all *p*s > 0.05 (Fig. 5D). Serum iron levels did not differ in the OWV and OEV groups versus the LWV and LEV groups, respectively, both with *p* > 0.05 (Fig. 5E). However, they were significantly elevated in the OWH (124.6%) versus OWV with *p* = 0.033. The rats in the LWV (189.7%) and LEV (171.0%) groups had significantly higher serum EtOH levels than those in the OWV and OEV groups, with *p* = 0.011 and *p* = 0.012, respectively (Fig. 5F). The serum EtOH levels were significantly elevated in the LEL (202.2%), OWL (193.6%) and OEL (218.4%) groups as compared to the LEV, OWV, and OEV groups, with *p* = 0.029, *p* = 0.027, and 0.013, respectively, and further elevated in the OWH (500.1%) and OEH (558.9%) groups versus OWV and OEV groups with *p* = 0.006 and *p* = 0.002, respectively.

### Metabolic Enzymes

**Fig. 6:**
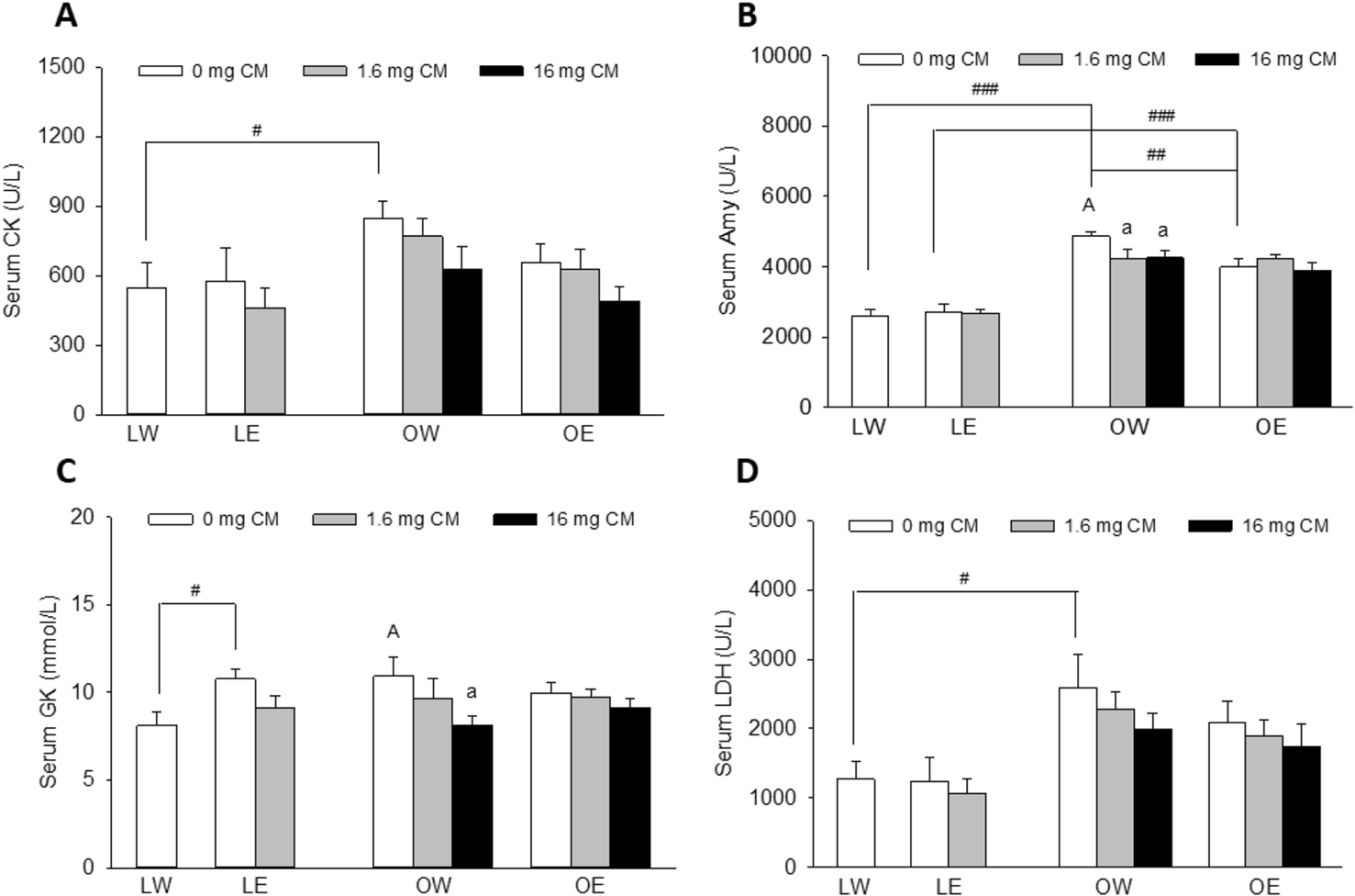
Effects of obesity, EtOH, and CM on markers of tissue injury and energy metabolism including serum creatine kinase (CK) (**A**), amylase (Amy) (**B**), glucokinase (GK) (**C**), lactate dehydrogenase (LDH) (**D**). Vertical bars represent the mean values from 4–8 rats. The error bars are the standard error of the means. “A” is significantly different from “a” at *p* < 0.05. “^#^”, “^##^”, and “^###^” indicate significant differences between the two treatment groups located under the vertical lines at *p* < 0.05, 0.01, and 0.001, respectively. *p* values were obtained from One Way ANOVA

The rats in the OWV (155.0%) group had significantly higher serum CK levels than those in the LWV group with *p* = 0.03 (Fig. 6A). A trend of decline in serum CK levels was observed in the OEV group versus the OWV group, and in the OWL, OWH, OEL and OEH groups related to their vehicle control group OWV and OEV groups, respectively, but the differences did not reach statistical significance levels with *p* > 0.05. The rats in the OWV (186.6%) and OEV (146.6%) groups had significantly higher serum amylase than those in the LWV and LEV groups, with *p* < 0.001 and *p* = 0.002, respectively. The rats in OEV (82.1%) group had significantly lower serum amylase than those in the OWV group with *p* = 0.008 (Fig. 6B). The rats in the OWL (86.9%) and OWH (87.4%) groups had significantly lower serum amylase levels than those in the OWV group with *p* = 0.046 and *p* = 0.021, respectively. The rats in the LEV (133%) group had significantly higher serum GK levels than those in the LWV group with *p* = 0.021 (Fig. 6C). The rats in the OWH (74.5%) group had significantly lower serum GK levels than those in the OWV group with *p* = 0.039. The rats in the OWV (204.2%) group had significantly higher serum LDH levels than those in the LWV group with *p* = 0.04 (Fig. 6D).

### Tissue Distribution and Concentration Factors of Mercury

Background tHg were detected in all tissues examined in a range of 1–1000 ppb (Table 2). Kidney had the highest tHg concentrations, followed by liver, pancreas, muscles, brain tissues, and serum. In all tissues, tHg concentrations increased with CM dose, but not proportionally. tHg concentrations in all tissues were significantly higher in the OEL than LEL group. tHg concentrations in the serum, liver, and muscle, but not in kidney, pancreas, hypothalamus/thalamus, cerebellum, cerebral cortex, and corpus callosum, were significantly higher in the OEV than LEV group.

**Table 2:** Effects of obesity, EtOH, and CM on tissue total mercury concentrations (mg/kg) shown as mean ± standard deviation

Treatment group	LEV	LEL	OWV	OWL	OWH	OEV	OEL	OEH
Serum (mg/L)	0.001 ± 0.000^A, D^	0.045 ± 0.017^aa, E^	0.008 ± 0.001^B^	0.225 ± 0.083^bbb^	0.727 ± 0.247^bbb^	0.005 ± 0.006^C, d^	0.187 ± 0.113^ccc, ee^	0.604 ± 0.390^ccc^
Liver*	0.042 ± 0.011^A, D^	1.275 ± 0.794^aaa, E^	0.063 ± 0.042^B, F^	3.895 ± 1.956^bbb^	8.728 ± 3.107^bbb^	0.530 ± 0.199^C, ddd, fff^	3.775 ± 1.287^ccc, ee^	11.94 ± 9.678^ccc^
Kidney	0.377 ± 0.136^A^	9.447 ± 3.832^aaa, D^	0.986 ± 0.887^B^	21.61 ± 12.37^bbb^	60.63 ± 25.06^bbb^	0.742 ± 0.538^C^	23.61 ± 8.726^ccc, ddd^	52.36 ± 22.93^ccc^
Muscle	0.027 ± 0.013^A, D^	1.118 ± 0.537^aa, E^	0.031 ± 0.024^B, F^	2.470 ± 1.461^bbb^	7.919 ± 2.194^bbb^	0.082 ± 0.090^C, ddd, f^	2.208 ± 0.747^ccc, e^	7.096 ± 3.109^ccc^
Pancreas	0.041 ± 0.013^A^	1.200 ± 0.744^aa, D^	0.049 ± 0.055^B^	3.770 ± 1.878^bbb^	12.40 ± 6.940^bbb^	0.058 ± 0.064^C^	3.331 ± 2.034^ccc, d^	10.94 ± 6.470^ccc^
Hypothalamus and thalamus	0.018 ± 0.008^A^	0.572 ± 0.261^aa, D^	0.017 ± 0.016^B^	1.238 ± 0.491^bbb^	4.088 ± 1.813^bbb^	0.035 ± 0.030^C^	1.249 ± 0.557^ccc, d^	3.463 ± 1.475^ccc^
Cerebellum	0.012 ± 0.005^A^	0.668 ± 0.238^aa, D^	0.018 ± 0.014^B^	1.418 ± 0.516^bbb^	4.989 ± 2.254^bbb^	0.032 ± 0.025^C^	1.395 ± 0.928^ccc, d^	4.416 ± 1.669^ccc^
Cerebral cortex	0,020 ± 0.019^A^	0.699 ± 0.400^aaa, D^	0.018 ± 0.016^B^	1.384 ± 0.948^bbb^	4.868 ± 1.375^bbb^	0.040 ± 0.029^C^	1.352 ± 0.498^ccc, d^	3.487 ± 1.224^ccc^
Corpus callosum	0.020 ± 0.010^A^	0.644 ± 0.271^aa, D^	0.019 ± 0.014^B^	1.459 ± 0.956^bbb^	5.070 ± 2.173^bbb^	0.034 ± 0.026^C^	1.209 ± 0.516^ccc, d^	3.815 ± 1.897^ccc^

Comparison was made between treatment groups for the same tissue. “A” is significantly different from “aa”, and “aaa” at *p* < 0.01 and 0.001, respectively. “B” is significantly different from “bbb” at *p* < 0.001. “C” is significantly different from “ccc” at *p* < 0.001. “D” is significantly different from “d” and “ddd” at *p* < 0.05 and 0.001, respectively. “E” is significantly different from “e” and “ee”, at *p* < 0.05 and 0.01, respectively. “F” is significantly different from “f” and “fff” at *p* < 0.05 and 0.001, respectively

*Values for OWV, OWH, OEV, and OEH were presented in Mailloux et al. [43]

Concentration factors (CFs) of tHg varied dramatically among organs (Table 3), with the highest found in kidney, followed by liver, pancreas, muscle, and brain tissues. tHg CFs in the liver were many folds higher for rats in the OEV group than the OWV group, and also significantly higher than those in the LEV group. The rats in the LEV group had significantly higher CFs in the kidney, pancreas, hypothalamus/thalamus, cerebellum, cerebral cortex, and corpus callosum, but not liver and muscle, than those in the OEV group. The rats in the LEL group had significantly higher CFs in the kidney, muscle, and all four brain tissues than those in the OEL group. The rats in the OEV had significantly higher CFs in the liver, muscle, hypothalamus/thalamus cerebral cortex, and corpus callosum than those in the OWV group.

**Table 3:** Effects of obesity, EtOH, and CM on tissue concentration factors (CFs) of total mercury content shown as mean ± standard deviation

Treatment Group	LEV	OWV	OEV	LEL	OWL	OEL
Liver	44.8 ± 9.3^a^	15.7 ± 3.2^aaa^	23.2 ± 242.5^A^	34.7 ± 5.6	17.7 ± 6.6	24.6 ± 10.4
Kidney	416.2 ± 150.6^bbb, oo^	164.0 ± 78.5	185.8 ± 81.2^B^	220.1 ± 59.1^h, O^	112.5 ± 35.0	145.2 ± 42.2^H^
Muscle	28.8 ± 9.7	9.0 ± 2.2^ccc^	20.1 ± 16.3^C^	25.1 ± 9.1^j^	10.9 ± 3.8	14.3 ± 5.2^J^
Pancreas	45.6 ± 14.3^ddd^	8.2 ± 6.8^p^	10.9 ± 4.5^D, s^	33.9 ± 9.1	17.0 ± 5.7^P^	25.3 ± 11.0^S^
Hypothalamus and thalamus	20.2 ± 9.3^e^	3.4 ± 1.8^e, q^	10.4 ± 7.0^E^	13.0 ± 3.5^kk^	5.8 ± 2.3^Q^	7.7 ± 2.8^K^
Cerebellum	12.8 ± 5.3	3.4 ± 2.1^r^	9.6 ± 9.9	15.6 ± 4.5^l^	6.8 ± 2.7^R^	8.2 ± 3.2^L^
Cerebral cortex	25.2 ± 29.5	4.0 ± 2.6^f^	12.7 ± 11.0^F^	15.0 ± 3.6^m^	6.7 ± 2.5	9.0 ± 4.9^M^
Corpus callosum	22.8 ± 14.2^g^	4.2 ± 2.2^g^	10.7 ± 12.1^G^	14.4 ± 2.3^nnn^	6.4 ± 2.9	7.7 ± 3.0^N^

Comparison was made between treatment groups for the same tissue. “A” is significantly different from “a” and “aaa” at *p* < 0.05 and 0.001, respectively. “B” is significantly different from “bbb” at *p* < 0.001. “C” is significantly different from “ccc” at *p* < 0.001. “D” is significantly different from “ddd” at *p* < 0.001. “E” is significantly different from “e” at *p* < 0.05. “F” is significantly different from “f” at *p* < 0.05. “G” is significantly different from “g” at *p* < 0.05. “H” is significantly different from “h” at *p* < 0.05. “J” is significantly different from “j” at *p* < 0.05. “K” is significantly different from “kk” at *p* < 0.01. “L” is significantly different from “l” *p* < 0.05. “M” is significantly different from “m” at *p* < 0.05. “N” is significantly different from “nnn” at *p* < 0.001. “O” is significantly different from “oo” at *p* < 0.01. “P” is significantly different from “p” at *p* < 0.05. “Q” is significantly different from “q” at *p* < 0.05. “R” is significantly different from “r” at *p* < 0.05. “S” is significantly different from “s” at *p* < 0.05

### Correlations Among CM Dose, Tissue tHg Contents and Clinical Parameters

To determine if CM doses and levels of tissue mercury, the most toxic component of the CM, had any positive or negative correlations with any parameters examined in this study, and if these correlations were altered by obesity and/or EtOH exposure, a Pearson Product Moment correlation analysis or Spearman Rank Order Correlation analysis was conducted for all parameters. The results revealed that tHg concentrations in all tissues examined were positively correlated with CM dose, regardless of obesity and EtOH exposure (Tables 4, S2–4). tHg concentrations in all tissues examined significantly and positively correlated to each other, regardless of obesity and EtOH treatment, except that the correlation between pancreas and cerebral cortex in the lean rats given EtOH did not reach statistically significance (supplemental Table S2).

**Table 4:** Major significant correlations found between contaminant mixture dose and tissue tHg concentrations and health parameters examined in rats of LEVL, OWVL, and OEVL groups

Treatment group	Health Parameters	CMD	Serum tHg	Liver tHg	Muscle tHg	Kidney tHg	Pancreas tHg	Hypothalamus and thalamus tHg	Cerebellum tHg	Cerebral Cortex tHg	Corpus Callosum tHg
LEVL	EtOH	**0.829**	NC	**0.786**	**0.824**	**0.835**	**0.793**	**0.807**	**0.811**	NC	**0.726**
	N/L-C	NC	**0.581**	**0.626**	**0.695**	**0.637**	NC	**0.636**	NC	NC	NC
	MCV	NC	*− 0.641*	*NC*	*− 0.58*	*− 0.794*	*− 0.793*	*− 0.687*	NC	NC	*− 0.606*
OWVL	W/E-C	*− 0.781*	*− 0.755*	*− 0.689*	*− 0.709*	*− 0.661*	*− 0.67*	*− 0.636*	*− 0.708*	*− 0.685*	*− 0.759*
	F-C	*− 0.792*	*− 0.777*	*− 0.79*	*− 0.803*	NC	*− 0.828*	*− 0.79*	*− 0.766*	*− 0.772*	*− 0.633*
	TG	*− 0.645*	*− 0.613*	*− 0.633*	*− 0.579*	*− 0.661*	*− 0.558*	NC	*− 0.655*	*− 0.507*	*− 0.510*
	Cl	**0.573**	**0.615**	**0.572**	**0.581**	**0.618**	**0.571**	**0.553**	**0.595**	**0.593**	**0.622**
	RDW	*− 0.581*	*− 0.627*	*− 0.64*	*− 0.616*	*− 0.571*	*− 0.569*	*− 0.574*	*− 0.574*	NC	NC
OEVL	F-C	*− 0.879*	NC	*− 0.804*	NC	*− 0.718*	*− 0.777*	*− 0.74*	*− 0.801*	*− 0.799*	*− 0.77*
	PON1	*− 0.883*	NC	*− 0.792*	NC	*− 0.753*	NC	*− 0.758*	*− 0.819*	*− 0.795*	*− 0.803*
	ALP	NC	**0.644**	**0.648**	NC	**0.571**	**0.701**	**0.666**	**0.768**	**0.567**	**0.666**
	BUN	NC	**0.702**	**0.594**	**0.577**	**0.57**	NC	**0.629**	NC	**0.618**	**0.518**
	EtOH	**0.816**	**0.853**	**0.842**	**0.898**	**0.819**	NC	**0.836**	**0.836**	**0.878**	**0.877**
	Mg	NC	*− 0.583*	*− 0.53*	NC	*− 0.502*	*− 0.507*	*− 0.552*	*− 0.528*	*− 0.532*	*− 0.519*
	MCP-1	NC	**0.509**	**0.564**	NC	**0.53**	**0.572**	**0.542**	**0.744**	**0.499**	**0.657**
	MCH	*− 0.557*	NC	*− 0.500*	*− 0.596*	*− 0.531*	NC	NC	NC	NC	*− 0.513*

Values are correlation coefficients with *p* < 0.05

Italic values indicate significant negative correlations. Bolded values indicate significant positive correlations

*LEVL* for lean rats given EtOH and treated with either vehicle or low-dose CM, *OWVL* for obese rats given water and treated with either vehicle or low-dose CM, *OEVL* for obese rats given EtOH and treated with either vehicle or low-dose CM, *CMD* for contaminant mixture dose, *EtOH* for serum ethanol, *N/L-C* for ratio of neutrophil to lymphocyte counts, *MCV* for mean corpuscular volume, *TG* for serum triglyceride, *Cl* for serum chloride, *RDW* for red cell distribution width, *F-C* for food consumption, *PON1* for serum paraoxonase-1, *ALP* for serum alkaline phosphatase, *BUN* for blood urea nitrogen, *Mg* for magnesium, *MCP-1* for monocyte chemotactic protein-1, *MCH* for mean corpuscular hemoglobin, *NC* indicates no significant correlations

Correlations between CM dose, tissue tHg and health parameters differed between lean and obese rats, and between rats given water and EtOH (Table 4). In the LEVL group, a significant positive correlation was found between tHg in all tissues except serum and cerebral cortex and serum EtOH levels, while negative correlations were found for N/L-C and MCV. In the OWVL group, however, a significant positive correlation was found for serum Cl, while negative correlations were found for W/E-C, F-C, TG, and RDW. In the OEVL group, positive correlations were found for ALP, BUN, EtOH, and MCP-1, and negative correlations for F-C, PON1, Mg, and MCH. Correlations were also found for other parameters as shown in Tables S2–4.

## Discussion

In our previous study, we examined the interaction of CM, EtOH, and obesity on parameters related to liver and pancreas health [43, 44]. In this study, we further determined the interplay of CM, EtOH, and obesity on the clinical biochemistry, physiology, hematology, histology, and some specific biomarkers related to CMD in JCR rats.

In the absence of EtOH and contaminants, the obese JCR rats had mild degree of heart histological lesions with focal cardiomyocyte necrosis and inflammatory cell infiltration that were similar to what was described for myocardial infarction in humans [47], which were not observed in the lean JCR rats. These obese rats were hyperlipidemic with several fold higher serum levels of TG, TC, LDL-C, and HDL-C than the normal lean rats, which is consistent with the typical serum lipid profile associated with obesity in humans [48, 49]. The obese rats also had an elevated systemic inflammation as shown by significantly higher NC, N/L-C, PLT, MPV, and serum CRP, and MCP-1 levels than the lean rats. This is in coherence with the inflammatory status associated with obesity found in humans [50, 51]. In addition, the obese rats had higher RBC and RDW, and lower MCV than the lean rats, which has also been found in humans with higher BMI [52] and waist circumference [52, 53], hypertension [54], insulin resistance [52], obesity and type 2 diabetes (T2D) [55].

The obese rats also displayed low serum creatinine, which may be attributed to low muscle mass as what is found in humans [56–58]. In fact, serum creatinine was also found to progressively decrease with obesity and T2D in nonhuman primates [59]. The lower serum creatinine levels in the obese rats paralleled with the decrease in serum Cl, but not Na, and the increase in serum UA. Low serum Cl is common in patients with chronic heart failure and is associated with worse outcomes [60, 61]. Cl is known to play an important role in regulating blood pressure [62, 63]. Cl depletion has been shown to stimulate renin secretion in the macula densa resulting in increased systemic blood pressure. UA has been shown to play a crucial role in pathogenesis of hypertension through potentially multiple mechanisms including upregulation of the renin–angiotensin–aldosterone system and increased oxidative stress and inflammation [64] Hyperuricemia was associated with metabolic syndrome [65] and increased risk for hypertension [66]. With the low serum Cl, high serum UA, and high water intake, the obese JCR rats would likely develop hypertension. Interestingly, in parallel with these changes, the obese JCR rats also expressed higher levels of serum 6-keto-PGF1α (hydrolytic product of prostacyclin), and NO, two key vessel dilators [67], which might have been activated as a compensatory mechanism to stabilize blood pressure. The higher circulating levels of 6-keto-PGF1α in the obese rats is consistence with its role in adipogenesis and obesity development [68]. All aforementioned observations provided experimental evidence for an increased risk of CVD associated with obesity.

Changes in serum aminotransferases levels have been found in many disease conditions in humans with NAFLD being most common [69, 70]. The elevated serum ALT, AST, ALP and TP levels in the obese JCR rats is consistent with the presence of the NAFLD observed in our previous study under the same experimental conditions [43], suggesting that hepatosteatosis associated with obesity is accompanied by liver damage, leading to increased serum protein levels and altered protein profiles. Changes in serum protein levels and profiles may affect blood viscosity, resulting in increased cardiovascular risks, as what is found in NAFLD patients [71, 72]. Elevated serum ALT levels have been linked to increased cardiovascular and metabolic risks in humans [73]. Disruption of the intestinal microbiome and the related metabolites are believed to contribute to the onset and progression of NAFLD [74–76]. Higher plasma EtOH were also found in children with NAFLD, and positively correlated with insulin, leptin, and TG [77]. In ob/ob mice, elevated plasma EtOH was attributed to decreased expression of cytochrome P450 2E1 and alcohol dehydrogenase in the liver as compared to lean control mice [77]. It is intriguing that, in this study, the obese JCR rats with hepatosteatosis had lower serum EtOH than the lean rats, while exposure to EtOH did not affect serum EtOH. It is possible that the obese JCR rats have different microbiome and/or EtOH metabolism from human NAFLD patients and ob/ob mice, although more investigations are warranted to clarify this.

Serum CK activity reflects levels of muscle damage and liver clearance of CK, and has been positively associated with blood pressure in some human populations [78, 79]. Elevated serum CK has been shown to predict first-ever myocardial infarction in Japanese populations [80]. This is coherent with the markedly increased serum CK detected in the obese JCR rats, suggesting that liver and muscle damage associated with obesity may contribute to the risk of CVD. Being mainly expressed in pancreas and liver, GK plays a primary role in regulating blood glucose homeostasis through affecting insulin release from pancreatic cells, and glycogen synthesis [81] and lipogenesis [82] in hepatocytes. Compromised liver and β-cell GK function and expression has been associated with patients with T2D, and GK activation has been shown to be effective in treating T2D [82]. Elevated GK activity in the obese JCR rats might prevent hyperglycemia, but would increase lipogenesis and lipid accumulation in the liver [43]. LDH is highly expressed and plays a major role in the production of lactate during anaerobic glycolysis in adipose tissue. Lactate released from adipose tissue is the main substrate for lipogenesis in the liver as a way to control glycemia in response to excess glucose level in the circulation [83]. Increases in serum ALT and LDH levels paralleled the increases in the hepatic ratios of alanine/pyruvate and lactate/pyruvate, respectively, in a rat model of NAFLD, suggesting their involvement in NAFLD [84]. The elevated serum LDH in the obese JCR rats is consistent with its positive correlation with obesity and fatty liver found in humans [85, 86].

In this study, the obese JCR rats had significantly higher tHg concentrations in serum, liver, and kidney than the lean rats, when they were exposed to background contaminant levels present in the vehicle group. When they were exposed to the low-dose CM, the obese rats accumulated higher levels of total mercury in all tissues examined than the lean rats. This could be attributed to both higher intake of mercury from food and water, and lower detoxification and excretion capability of the obese than the lean rats, suggesting that obese individuals may be prone to higher mercury accumulation. This is consistent with the reported link between mercury exposure, obesity and obesity related clinical profile in some human populations [87–90]. However, it remains to be established if mercury exposure caused obesity and related health effects, or whether obesity and related health deficits resulted in higher retention of mercury. Obesity not only affected tissue mercury levels, but also altered tissue distribution of mercury, rendering higher proportion of mercury distributed in the liver and less in kidney, pancreas, and brain tissues, when compared to lean rats. This could be attributed to the increased fat accumulation in the liver of obese rats.

The results of this and our previous studies suggest that obesity has a broad impact on the physiological- and organ-specific parameters. In contrast, the influence of EtOH on these parameters is more specific and different in lean from obese rats. In our previous study [43], we found that in the obese rats, EtOH increased hepatic expression of geranylgeranyl diphosphate synthase and diphosphomevalonate decarboxylase involved in cholesterol synthesis [43]. It also increased hepatic expression of cytochrome P450 family 2 subfamily E member 1 (CYP2E1), an enzyme known to be involved in EtOH metabolism and oxidative stress, and elevated in obesity and hepatosteatosis in humans [91]. In addition, EtOH decreased hepatic expression of ATP5A, a subunit of mitochondrial ATP synthase involved in mitochondrial ATP production [43]. These changes in liver observed from our previous study paralleled with decreases in serum TG, TC, LDL-C, BUN, UA, TP, and ALP observed in this study, suggesting that EtOH exposure disrupted hepatic mitochondrial energy production, lipid metabolism, urea synthesis and/or secretion, and metabolism of purine bases, complicating clinical profiles and diagnosis. Although the decreased serum TG, TC, and LDL-C by EtOH exposure may lessen the risk for atherosclerosis in the obese rats, the decreased serum 6-keto-PGF1α and NO in the obese rats exposed to EtOH may compromise the capability of obese rats to regulate blood pressure, increasing the risk for hypertension.

The effects of CM in obese rats were mostly suppressive and/or damaging. In our previous studies [43], we found that obesity in JCR rats were associated with hepatosteatosis, which was exacerbated by exposure to ethanol and/or CM. While ethanol increased protein levels of some enzymes involved in cholesterol synthesis in the liver, CM decreased the levels of the same and other enzymes involved in cholesterol synthesis in the presence and absence of ethanol, suggesting that CM exposure counteracted on the action of ethanol in the liver. CM, but not ethanol, decreased expression of proteins involved in lipid-binding (liver fatty acid binding protein, LFABP) and transport (ATP-binding cassette transporter A1, ABCA1) in the liver, which paralleled with decreases in serum TG, TC, LDL-C and HDL-C by CM exposure observed in this study. This suggests that CM exacerbated hepatosteatosis by increasing lipid synthesis and/or decreasing lipid and/or lipoprotein export from liver to circulation. This also suggest that different mechanisms may be involved in the effects of ethanol from those of CM on hepatosteatosis. The decreased serum lipid and lipoprotein by high-dose CM may relieve the risk for atherosclerosis, however, can also increase the chance for misdiagnosis and overlooking of the hepatosteatosis associated with obesity. The increased serum CRP, an acute response protein release from liver known to be associated with CVD, and the decreased serum PON1, an antioxidant enzyme protecting HDL-C from oxidation, ApoA1, a HDL-C associated protein known to play an role in maintaining HDL-C function, and 6-keto-PGF1α, a non-enzymatic hydrolysis product of prostacyclin that functions as vessel dilator, by high-dose CM in the obese rats suggest a further increase in the risk for CVD and hypertension in these rats. In addition to heart and liver injuries, CM also disrupted pancreatic structure and function, causing decreased α and β cell mass, increased pancreatic iron deposit and monocyte infiltration, and decreased production and release of insulin and glucagon in obese rats [44]. These changes paralleled with elevated serum iron and islet cell antibodies and decreased serum amylase and glutamic acid decarboxylase autoantibody. These results suggest that CM exposure may promote the progression of disease state from features of T2D to features of T1D in the obese rats. The most intriguing finding of this study was that both low- and high-dose CM increased serum EtOH levels in both lean and obese rats with or without co-exposure with EtOH. This could be attributed to increased endogenous production of EtOH from microflora or decreased metabolism of EtOH, although more studies are warranted to confirm this. In fact, contaminants have been shown to affect gut microflora, while microflora can also affect metabolism of contaminants [92, 93]. High-alcohol-producing Klebsiella pneumoniae (HiAlc Kpn) was associated with up to 60% of individuals with NAFLD in a Chinese cohort [94]. In this study, CM exacerbated the hepatosteatosis associated with obesity. However, the sources and health consequences resulted from CM-induced increase in endogenous EtOH observed in JCR rats remain to be investigated.

**Fig. 7:**
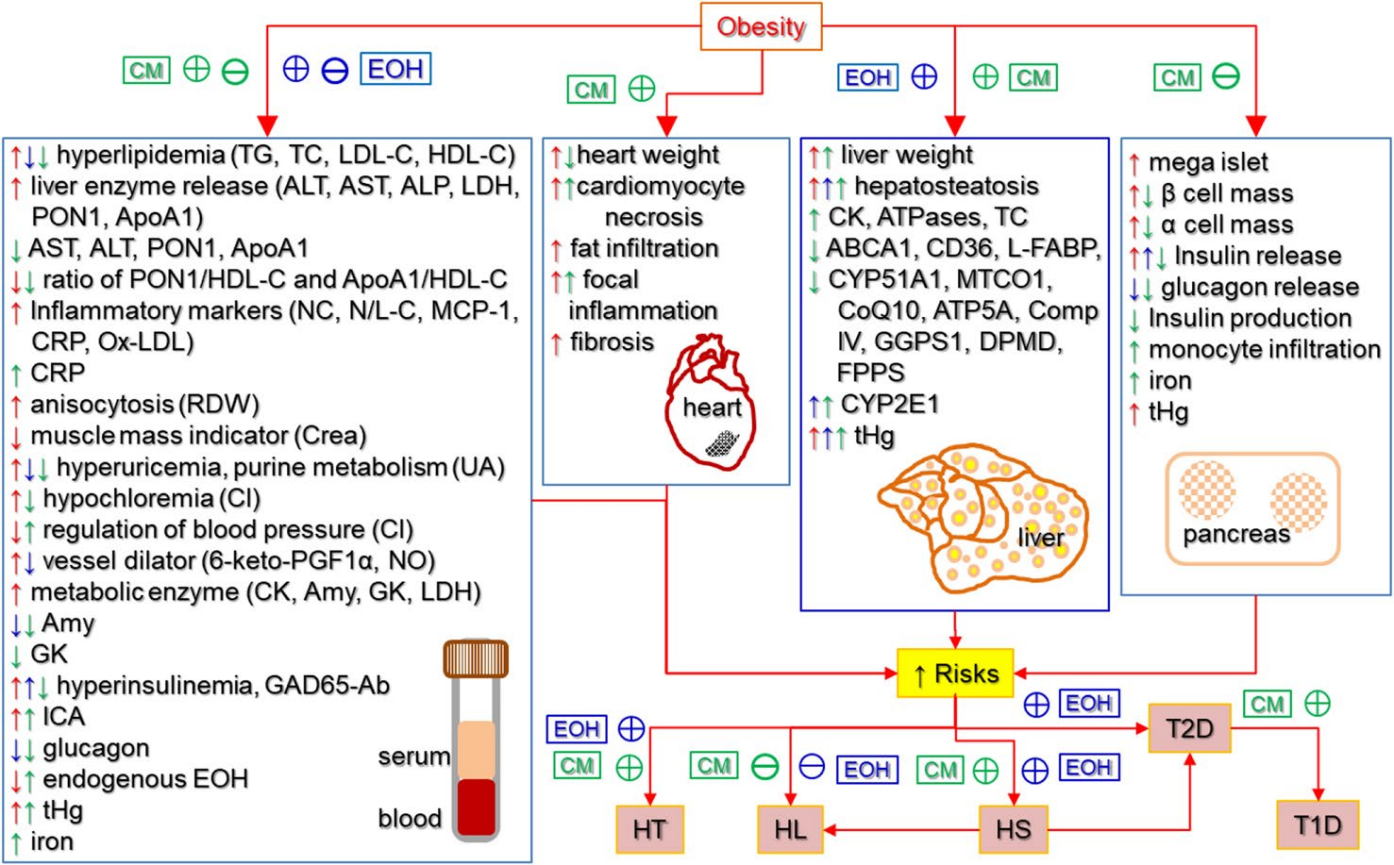
Summary of the effects of obesity, EtOH, and CM and their interactions on organ and serum parameters. These parameters are related to risks of hyperlipidemia (HL), hypertension (HT), hepatosteatosis (HS), and diabetes (T1D, T2D) in JCR rats observed in our previous and current studies. Upward arrows indicate promoting or increasing effects. Downward arrows indicate inhibiting or decreasing effects. Red color is used for the effects of obesity. Blue color is used for the effects of EtOH. Green color is used for the effects of CM. Circles with plus symbols indicate promoting interaction or positive correlations while circles with minus symbols indicate inhibiting interaction or negative correlations. TG: triglyceride. *TC* total cholesterol, *LDL-C* low-density lipoprotein cholesterol, *HDL-C* high-density lipoprotein cholesterol, *ALT* alanine aminotransferase, *AST* aspartate aminotransferase, *ALP* alkaline phosphatase, *LDH* lactate dehydrogenase, *PON1* paraoxonase-1, *ApoA1* apolipoprotein A1, *NC* neutrophil counts, *N/L-C* ratio of neutrophil to lymphocyte counts, *MCP-1* monocyte chemoattractant protein-1, *CRP* C-reactive protein, *Ox-LDL* oxidized low-density lipoprotein, *RDW* red cell distribution width, *Crea* creatinine, *UA* uric acid, *CL* chloride, *6-keto-PGF1α* 6-keto-protaglandane F1 alpha, *NO* nitric oxide, *CK* creatinine kinase, *Amy* amylase, *GK* glucokinase, *GAD65-Ab* glutamic acid decarboxylase autoantibody, *ICA* islet cell antibody, *ATPases* adenosine triphosphatases, *ABCA1* ATP-binding cassette transporter 1, *CD36* fatty acid translocase or scavenger receptor class B member 3, *LFABP* liver fatty acid binding protein, *CYP51A1* cytochrome P450 family 51 subfamily A member 1, *MTCO1* mitochondrially encoded cytochrome c oxidase I, *CoQ10* co-enzyme Q10, *ATP5A* ATP synthase lipid-binding protein, *Comp IV* cytochrome c oxidase or Complex IV, *GGPS1* geranylgeranyl diphosphate synthase, *MVD* diphosphomevalonate decarboxylase, *FDPS* farnesyl pyrophosphate synthase, *CYP2E1* cytochrome P450 family 2 subfamily E member 1

In summary, findings from our previous and current studies suggested that in JCR rats, obesity was associated with clinical profiles and organ structural and functional changes that points to increased risk for CVD (Fig. 7). Obesity also increased the risk for contaminant-induced toxicity. Heavy exposure to EtOH exacerbated the state of NAFLD and hyperinsulinemia, while lessening the state of hyperlipidemia associated with obesity. In addition, EtOH exposure increased the risks of developing hypertension and liver intoxication by contaminants in obese rats. Heavy exposure to CM lessened or diminished hyperlipidemia and hyperinsulinemia, but intensified the hepatosteatosis associated with obesity. CM exposure worsened systemic inflammation and cardiomyocyte injury, while also increasing the risk for hypertension in obese rats. Combined exposure to EtOH and CM caused increased liver toxicity, and more complicated physiological profile with marker changes pointing to different health outcomes. MeHg may be responsible for the effects of low-dose CM on circulating TG, Cl, PON1, and EtOH levels. Endogenous EtOH production and metabolism may be a sensitive target of CM, which requires further investigation. Our studies demonstrated that JCR rats are very useful animal models for studying the effects of obesity, ethanol and/or exposure to chemical contaminants, and their interplay on biomarkers related human CMD. However, the human implications of our findings may be limited due to species differences in metabolism, nutritional status, and contaminant exposure levels, which should be considered in interpretation of the information generated from JCR rats. The high CM dose group used in this study represents a scenario that one is exposed to all the component chemicals at ten times of the highest level found in human blood of some highly exposed Northern populations, which is unlikely to happen in real world. Nonetheless, it serves as a positive control for studying the potential adverse health effects of CM.

## Electronic supplementary material

Below is the link to the electronic supplementary material.

Supplementary file1 (DOCX 43 KB)

Supplementary file2 (DOCX 15 KB)

Supplementary file3 (DOCX 29 KB)

Supplementary file4 (DOCX 26 KB)

Supplementary file5 (DOCX 26 KB)

## Data Availability

The authors declare that all supporting data are available within the article and its online data supplement.

## Abbreviations

ALT: Alanine aminotransferase
Amy: Amylase
ApoA1: Apolipoprotein A1
ALP: Alkaline phosphatase
AST: Aspartate aminotransferase
Bil-D: Deconjugated bilirubin
Bil-T: Total bilirubin
BUN: Blood urea nitrogen
BW: Body weight
CCAC: Canadian Council on Animal Care
Cl: Chloride
CK: Creatine kinase
CM: Contaminant mixture
Crea: Creatinine
CRP: C-reactive protein,
CVDs: Cardiovascular diseases
CMDs: Cardiovascular and metabolic diseases
EtOH: Ethanol
F-C: Food consumption
GK: Glucokinase
HDL-C: High-density lipoprotein cholesterol
H/L-C: Ratio of high to low-density lipoprotein
HW: Heart weight
% HW: Heart weigh as percentage of body weight
6-keto-PGF: 6-Keto-prostaglandin F1α
LEV: Lean rats given 10% ethanol and treated with vehicle
LEL: Lean rats given 10% ethanol and treated with low-dose contaminant mixture
LWV: Lean rats given water and treated with vehicle
LC: Lymphocyte count
LDH: Lactate dehydrogenase
LDL-C: Low-density lipoprotein cholesterol
MCP-1: Monocyte chemotactic protein-1
MCV: Mean corpuscular volume
MeHg: Methylmercury
MPV: Mean platelet volume
NAFLD: Non-alcoholic fatty liver diseases
NC: Neutrophil count
N/L-C: Ratio of neutrophil to lymphocyte count,
NO: Nitric oxide
OEV: Obese rats given 10% ethanol and treated with vehicle
OEL: Obese rats given 10% ethanol and treated with low-dose contaminant mixture
OEH: Obese rats given 10% ethanol and treated with high-dose contaminant mixture
OEVL: Obese rat vehicle control and low-dose (1.6 mg/kg BW) groups given 10% EtOH
OWV: Obese rats given water and treated with vehicle
OWL: Obese rats given water and treated with low-dose contaminant mixture
OWH: Obese rats given water and treated with high-dose contaminant mixture
Ox-LDL: Oxidized low-density lipoprotein
P: Phosphate
PCBs: Polychlorinated biphenyls
PFOS: Perfluorooctane Sulfonate
PLT: Platelet count
PON1: Paraoxonase-1
PON1/HDL-C: Ratio of paraoxonase-1 to high-density lipoprotein cholesterol
tHg: Total mercury concentration
TG: Triglycerides
TC: Total cholesterol
T-Fe: Total iron
TP: Total protein
RDW: Red blood cell distribution width
UA: Uric acid
WBC: White blood cell
W/E-C: Water or ethanol consumption
