# Supplementary material for: Interplay of Obesity, Ethanol, and Contaminant Mixture on Clinical Profiles of Cardiovascular and Metabolic Diseases: Evidence from an Animal Study
Source: Cardiovasc Toxicol. 2022 Apr 16;22(6):558–78. doi: 10.1007/s12012-022-09738-6 (PMC9107407; doi:10.1007/s12012-022-09738-6)
Supplement: Supplementary file 1 — Supplementary file1 (DOCX 43 KB) [file 12012_2022_9738_MOESM1_ESM.docx]

**Appendix A: Supplementary Data**


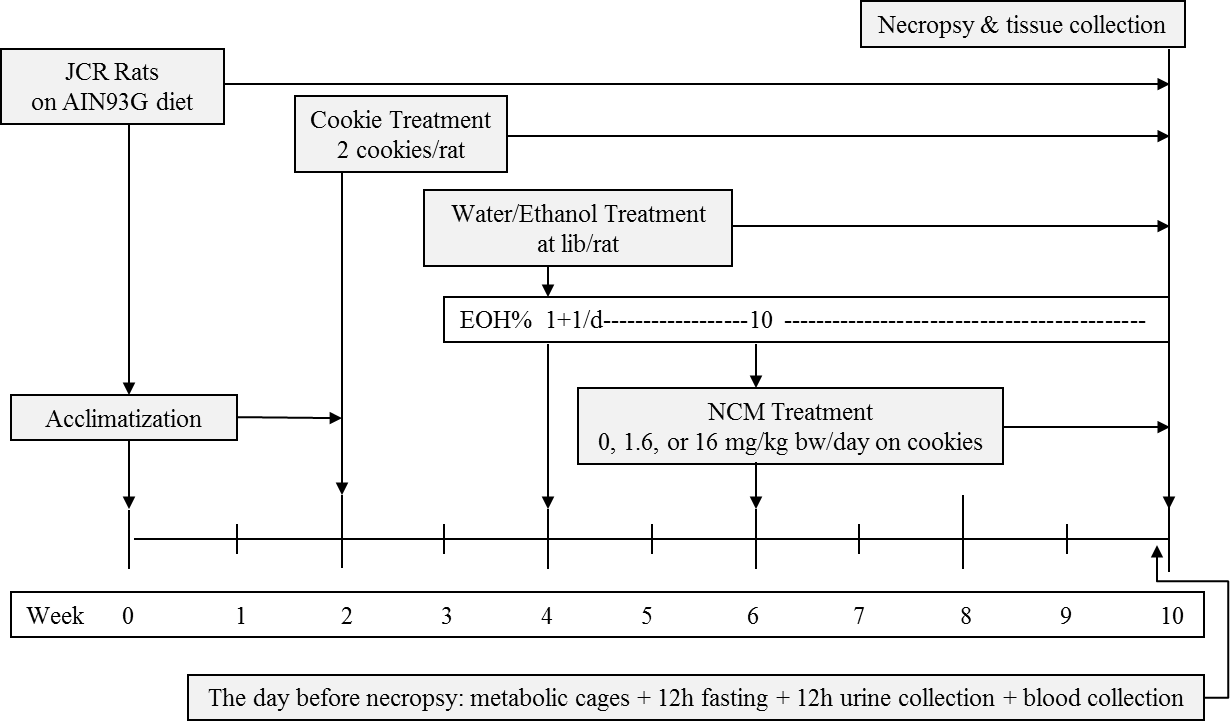


Figure S1. Experimental design and dosing scheme. 18 lean and 48 Obese rats at 8 weeks age were acclimatized on AIN93G diet for two weeks, followed by two cookies/day/rat treatment for two weeks. Then, the rats were separated into two groups, one given distilled and deionized water at lib, and one given 1-10% EtOH at lib for two weeks. For EtOH treatment, the rats were started with 1% EtOH in distilled and deionized water and followed by additional one more percentage alcohol per day until 10% EtOH. After two weeks of water or alcohol treatment, the rats were given water or 10% alcohol for four addition weeks during which they were also given two cookies loaded with CM at 0 (corn oil), 1.6, and 16 mg/kg BW/day. After 4 weeks of CM treatment, the rats were euthanized. Blood, serum and organs were collected and weighed.
