# Supplementary material for: Interplay of Obesity, Ethanol, and Contaminant Mixture on Clinical Profiles of Cardiovascular and Metabolic Diseases: Evidence from an Animal Study
Source: Cardiovasc Toxicol. 2022 Apr 16;22(6):558–78. doi: 10.1007/s12012-022-09738-6 (PMC9107407; doi:10.1007/s12012-022-09738-6)
Supplement: Supplementary file 2 — Supplementary file2 (DOCX 15 KB) [file 12012_2022_9738_MOESM2_ESM.docx]

| Table S1. CM chemical composition and concentrations detected in Northern Canadian blood or plasma and CM doses used in this study. | | | |
| --- | --- | --- | --- |
| CM chemical composition | Mean human blood or plasma level (µg/L) (Dewailley et al. 2006) | Chemical Dose | |
|  |  | Low dose (μg/kg BW)  (the highest concentration found in human blood or plasma) | High dose (μg/kg BW)  (10 times of the highest concentration found in human blood or plasma) |
| Heavy Metals (3) | | | |
| Cadmium | 3.035 (blood) | 146.10 | 1461.00 |
| Methylmercury | 10.997 (blood) | 241.00 | 2410.00 |
| Lead | 39.368 (blood) | 497.30 | 4972.80 |
| Polychlorinated biphenyls (PCBs) (11) | | | |
| 99 | 0.170 | 4.70 | 47.00 |
| 138 | 0.534 | 19.00 | 190.00 |
| 146 | 0.180 | 6.10 | 61.00 |
| 153 | 1.333 | 40.00 | 400.00 |
| 163 | 0.221 | 6.20 | 62.00 |
| 170 | 0.216 | 6.18 | 61.82 |
| 180 | 0.813 | 22.70 | 227.27 |
| 187 | 0.287 | 9.10 | 91.00 |
| 194 | 0.182 | 5.10 | 51.00 |
| 201 | 0.167 | 4.70 | 47.00 |
| 203 | 0.105 | 2.50 | 25.00 |
| Organochlorines (4) | | | |
| Oxychlordane | 0.431 | 16.00 | 160.00 |
| p,p’-DDE | 3.232 | 50.00 | 500.00 |
| Trans-nonachlor | 0.725 | 22.00 | 220.00 |
| Pentachlorophenol | 0.914 | 18.00 | 180.00 |
| Toxaphene (1) |  |  |  |
| Parlar # 50 | 0.142 | 59.10 | 59.13 |
| Brominated flame retardants (2) | | | |
| PBDE IUPAC #47 | 0.072 | 2.40 | 24.00 |
| 2,3,4,6-Tetrabromophenol | 0.036 | 1.39 | 13.95 |
| Perfluorinated compounds (1) | | | |
| PFOS | 29.000 | 470.00 | 4700.00 |
| Total concentrations | 101.929 | 1596.40 | 15963.97 |
