# Supplementary material for: Interplay of Obesity, Ethanol, and Contaminant Mixture on Clinical Profiles of Cardiovascular and Metabolic Diseases: Evidence from an Animal Study
Source: Cardiovasc Toxicol. 2022 Apr 16;22(6):558–78. doi: 10.1007/s12012-022-09738-6 (PMC9107407; doi:10.1007/s12012-022-09738-6)
Supplement: Supplementary file 4 — Supplementary file4 (DOCX 26 KB) [file 12012_2022_9738_MOESM4_ESM.docx]

| Table S3. Profile of significant correlations^†^ between NCM, total Hg, and clinical endpoints in OWVL groups | | | | | | | | | | | |
| --- | --- | --- | --- | --- | --- | --- | --- | --- | --- | --- | --- |
|  | **Endpoints** | **CMD** | **S-tHg** | **L-tHg** | **M-tHg** | **K-tHg** | **P-tHg** | **H/T-tHg** | **Ce-tHg** | **Co-tHg** | **CC-tHg** |
| **Total Hg content** | **S-tHg** | **0.886** |  |  |  |  |  |  |  |  |  |
|  | **L-tHg** | **0.829** | **0.888** |  |  |  |  |  |  |  |  |
|  | **M-tHg** | **0.784** | **0.895** | **0.984** |  |  |  |  |  |  |  |
|  | **K-tHg** | **0.782** | **0.821** | **0.951** | **0.958** |  |  |  |  |  |  |
|  | **P-tHg** | **0.832** | **0.91** | **0.962** | **0.979** | **0.932** |  |  |  |  |  |
|  | **H/T-tHg** | **0.883** | **0.883** | **0.928** | **0.92** | **0.849** | **0.951** |  |  |  |  |
|  | **Ce-Hg** | **0.899** | **0.867** | **0.973** | **0.938** | **0.933** | **0.943** | **0.946** |  |  |  |
|  | **C0-tHg** | **0.737** | **0.861** | **0.876** | **0.931** | **0.891** | **0.961** | **0.869** | **0.85** |  |  |
|  | **CC-Hg** | **0.751** | **0.872** | **0.892** | **0.937** | **0.913** | **0.935** | **0.883** | **0.877** | **0.955** |  |
| **Physiology** | **BW** | **0** | **0** | **0** | **0** | **0** | **0** | **0** | **0** | **0** | **0** |
|  | **W/E-C** | **-0.781** | **-0.755** | **-0.689** | **-0.709** | **-0.661** | **-0.67** | **-0.636** | **-0.708** | **-0.685** | **-0.759** |
|  | **F-C** | **-0.792** | **-0.777** | **-0.79** | **-0.803** | **0** | **-0.828** | **-0.79** | **-0.766** | **-0.772** | **-0.633** |
|  | **HW** | **0** | **0** | **0** | **-0.52** | **-0.532** | **-0.511** | **0** | **0** | **-0.521** | **-0.569** |
|  | **%HW** | **0** | **0** | **0** | **0** | **0** | **0** | **0** | **0** | **-0.56** | **0** |
| **Cardiovascular health** | **LDH** | **0** | **0** | **0** | **0** | **0** | **0** | **0** | **0** | **0** | **0** |
|  | **%Lip** | **0** | **0** | **0** | **0** | **0** | **0** | **0** | **0** | **0** | **0** |
|  | **TC** | **0** | **0** | **0** | **0** | **0** | **0** | **0** | **0** | **0** | **0** |
|  | **LDL-C** | **0** | **0** | **0** | **0** | **0** | **0** | **0** | **0** | **0** | **0** |
|  | **HDL-C** | **0** | **-0.485** | **0** | **0** | **-0.528** | **0** | **0** | **0** | **0** | **0** |
|  | **H/L-C** | **0** | **0** | **0** | **0** | **0** | **0** | **0** | **0** | **0** | **0** |
|  | **Lipa** | **0** | **0** | **0** | **0** | **0** | **0** | **0** | **0** | **0** | **0** |
|  | **TG** | **-0.645** | **-0.613** | **-0.633** | **-0.579** | **-0.661** | **-0.558** | **0** | **-0.655** | **-0.507** | **-0.51** |
|  | **UA** | **0** | **0** | **0** | **0** | **0** | **0** | **0** | **0** | **0** | **0** |
|  | **Ox-LDL** | **0** | **0** | **0** | **0** | **0** | **0** | **0** | **0** | **0** | **0** |
|  | **PON1** | **0** | **0** | **0** | **0** | **0** | **0** | **0** | **0** | **0** | **0** |
|  | **PON1/HDL-C** | **0** | **0** | **0** | **0** | **0** | **0** | **0** | **0** | **0** | **0** |
|  | **ApoA1** | **0** | **0** | **0** | **0** | **0** | **0** | **0** | **0** | **0** | **0** |
|  | **NO** | **-0.514** | **0** | **0** | **0** | **-0.522** | **0** | **0** | **0** | **0** | **-0.525** |
|  | **6-keto-PGF** | **0** | **0** | **0** | **0** | **0** | **0** | **0** | **0** | **0** | **0** |
| **Liver function** | **Alb** | **0** | **0** | **0** | **0** | **0** | **0** | **0** | **0** | **0** | **0** |
|  | **TP** | **0** | **0** | **-0.552** | **-0.528** | **-0.529** | **0** | **0** | **0** | **0** | **0** |
|  | **ALT** | **0** | **0** | **0** | **0** | **0** | **0** | **0** | **0** | **0** | **0** |
|  | **AST** | **0** | **0** | **0** | **0** | **0** | **0** | **0** | **0** | **0** | **0** |
|  | **ALP** | **0** | **0** | **0** | **0** | **0** | **0** | **0** | **0** | **0** | **0** |
|  | **BUN** | **0** | **0** | **0** | **0** | **0** | **0** | **0** | **0** | **0** | **0** |
|  | **Bil-D** | **0** | **0** | **0** | **0** | **0** | **0** | **0** | **0** | **0** | **0** |
|  | **Bil-T** | **0** | **0** | **0** | **0** | **0** | **0** | **0** | **0** | **0** | **0** |
|  | **EtOH** | **0.764** | **0.864** | **0** | **0** | **0** | **0** | **0** | **0** | **0** | **0.739** |
| **Kidney function** | **Cl** | **0.573** | **0.615** | **0.572** | **0.581** | **0.618** | **0.571** | **0.553** | **0.595** | **0.593** | **0.622** |
|  | **Na** | **0** | **0** | **0** | **0** | **0** | **0** | **0** | **0** | **0** | **0** |
|  | **K** | **0** | **0** | **0** | **0** | **0** | **0** | **0** | **0** | **0** | **0** |
|  | **Ca** | **0** | **0** | **0** | **0** | **0** | **0** | **0** | **0** | **0** | **0** |
|  | **P** | **0** | **0** | **0** | **0** | **-0.581** | **0** | **0** | **-0.52** | **0** | **0** |
|  | **Mg** | **0** | **0** | **0** | **0** | **0** | **0** | **0** | **0** | **0** | **0** |
|  | **Cre** | **0** | **0** | **0** | **0** | **0** | **0** | **0** | **0** | **0** | **0** |
| **Metabolism** | **LDH** | **0** | **0** | **0** | **0** | **0** | **0** | **0** | **0** | **0** | **0** |
|  | **CK** | **0** | **0** | **0** | **0** | **0** | **0** | **0** | **0** | **0** | **0** |
|  | **GK** | **0** | **0** | **0** | **0** | **0** | **0** | **0** | **0** | **0** | **0** |
|  | **Amy** | **0** | **0** | **-0.521** | **-0.516** | **-0.54** | **0** | **0** | **0** | **0** | **0** |
| **Inflamation and immune response** | **CRP** | **0** | **0** | **0** | **0** | **0** | **0** | **0** | **0** | **0** | **0** |
|  | **MCP-1** | **0** | **0** | **0** | **0** | **0** | **0** | **0** | **0** | **0** | **0** |
|  | **T-Fe** | **0** | **0** | **0** | **0** | **0** | **0** | **0** | **0** | **0** | **0** |
|  | **WBC** | **0** | **0** | **0** | **0** | **0** | **0** | **0** | **0** | **0** | **0** |
|  | **NC** | **0** | **0** | **0** | **-0.535** | **0** | **0** | **0** | **0** | **0** | **0** |
|  | **LC** | **0** | **0** | **0** | **0** | **0** | **0** | **0** | **0** | **0** | **0** |
|  | **N/L-C** | **0** | **0** | **0** | **0** | **0** | **0** | **0** | **0** | **0** | **0** |
|  | **MC** | **0** | **0** | **0** | **0** | **0** | **0** | **0** | **0** | **0** | **0** |
|  | **N/M-C** | **0** | **0** | **0** | **0** | **0** | **0** | **0** | **0** | **0** | **0** |
|  | **EC** | **0** | **0** | **0** | **0** | **0** | **0** | **0** | **0** | **0** | **0** |
|  | **N/E-C** | **0** | **0** | **-0.542** | **-0.545** | **0** | **-0.521** | **0** | **0** | **0** | **0** |
|  | **BC** | **0** | **0** | **0** | **0** | **0** | **0** | **0** | **0** | **0** | **0** |
|  | **N/B-C** | **0** | **0** | **0** | **0** | **0** | **0** | **0** | **0** | **0** | **0** |
|  | **PLT** | **0** | **0** | **0** | **0** | **0** | **0** | **0** | **0** | **0** | **0** |
|  | **MPV** | **-0.558** | **0** | **0** | **0** | **0** | **0** | **0** | **0** | **0** | **0** |
|  | **RBC** | **0** | **0** | **0** | **0** | **0** | **0** | **0** | **0** | **0** | **0** |
| **Red blood cells** | **HGB** | **0** | **0** | **0** | **0** | **0** | **0** | **0** | **0** | **0** | **0** |
|  | **HCT** | **0** | **0** | **0** | **0** | **0** | **0** | **0** | **0** | **0** | **0** |
|  | **MCV** | **0** | **0** | **0** | **0** | **0** | **0** | **0** | **0** | **0** | **0** |
|  | **MCH** | **0** | **0** | **0** | **0** | **0** | **0** | **0** | **0** | **0** | **0** |
|  | **MCHC** | **0** | **0** | **0** | **0** | **0** | **0** | **0** | **0** | **0** | **0** |
|  | **RDW** | **-0.581** | **-0.627** | **-0.64** | **-0.616** | **-0.571** | **-0.569** | **-0.574** | **-0.574** | **0** | **0** |

“Red”, “yellow”, and “green” colours indicate significant positive, insignificant, and significant negative correlations, between the two parameters at p< 0.05, respectively. “0” indicates no significant correlation, and thus the values of correlation coefficients were omitted. Values except 0 are Pearson Product Moment correlation coefficients. OWVL: Obese rat vehicle control and low dose (1.6 mg/kg BW) groups given water. CMD: contaminant mixture dose. tHg: total mercury concentration, S: serum, L: liver, M: muscle, K: kidney, P: pancreas, H/T: hypothalamus/thalamus, Ce: cerebellum, Co: cerebral cortex, CC: corpus callosum, BW: body weight, W/E-C: water or EtOH consumption, F-C: food consumption, HW: heart weight, % HW: percentage heart weigh, LDH: low density lipoprotein, %Lip: percentage lipid content by weight, TC: total cholesterol, LDL-C: low density lipoprotein-cholesterol, HDL-C: high density lipoprotein cholesterol, H/L-C: ratio of high to low density lipoprotein, Lipa: lipase, TG: triglycerides, UA: uric acid, Ox-LDL: oxidized low density lipoprotein, PON1: paroxonase-1, PON1/HDL-C: ratio of paoxonase-1 to high density lipoprotein cholesterol, ApoA1: apolipoprotein A1, NO: nitric oxide, 6-keto-PGF: 6-keto prostaglandin F1α, TP: total protein, ALT: alanine aminotransferase, AST: aspartate aminotransferase, ALP: alkaline phosphatase, BUN: blood urea nitrogen, Bil-D, deconjugated bilirubin, Bil-T: total bilirubin, EtOH: ethanol, Cl: chloride, Na: sodium, K: potassium, Ca: calcium, P: phosphate, Mg: magnesium, Cre: creatinine, Adip: adiponectin, CK: creatine kinase, GK: glucokinase, Amy: amylase, CRP : C-reactive protein, MCP-1 : monocyte chemotactic protein-1, T-Fe : total iron, WBC : white blood cell, NC : neutrophil count, LC: lymphocyte count, N/L-C ratio of neutrophil to lymphocyte count, MC: monocyte count, N/M-C: ratio of neutrophil to monocyte count, EC: eosinophil count, N/E-C: ratio of neutrophil to eosinophil count, BC: basophil count, N/B-C: ratio of neutrophil to basophil count, PLT: platelet, MPV: mean platelet volume, RBC: red blood cell, HGB: hemoglobin, HCT: hematocrit, MCV: mean corpuscular volume, MCH: mean corpuscular hemoglobin, MCHC: mean corpuscular hemoglobin concentration, RDW: red blood cell distribution width.
